# Driver gene-specific prevalence and incidence of brain metastases in non-small cell lung cancer: a meta-analysis encompassing all disease stages

**DOI:** 10.1007/s12672-025-03621-w

**Published:** 2025-09-30

**Authors:** Yu Zhang, Yuxin Wen, Peiyang Fan, Fangfang Li, Muye Li, Zhiyuan Wang, Xiaofang Wang, Zichen Mao, Yanxun Li, Yuchen Yao, Li Bian

**Affiliations:** 1 Department of Pathology, The First Affiliated Hospital of Kunming Medical University, Kunming, Yunnan China; 2 School of Basic Medical Sciences, Kunming Medical University, Kunming, Yunnan China; 3 No.1 School of Clinical Medicine, Kunming Medical University, Kunming, China; 4 Department of Pathology, The Second Affiliated Hospital of Kunming Medical University, Kunming, Yunnan China

**Keywords:** Non-small cell lung cancer, Brain metastases, Incidence, Prevalence

## Abstract

**Background:**

Brain metastases (BM) in non-small cell lung cancer (NSCLC) are associated with a poor prognosis. Identifying relevant genomic alterations can facilitate the targeting of therapies. We conducted a systematic review and meta-analysis to assess the prevalence and incidence of these alterations in NSCLC patients.

**Methods:**

PubMed, Embase and WOS were searched, from January 2000 to February 2024 using "lung", "met" and "incidence" as search phrases. We obtained data on the prevalence at diagnosis and the annual incidence of new BM in patients with EGFR, ALK, KRAS, and other genetic alterations. The pooled prevalence and incidence rates were calculated using a random effects model. This study is registered with PROSPERO (CRD42023491178).

**Results:**

A total of 120 articles were included in the analysis. Prevalence data were derived from 94 studies (17,458 patients), while incidence data were obtained from 95 studies (13,323 patients). The pooled prevalence of BM at diagnosis was found to be 28.8% (95% [CI]: 0.263–0.313). This prevalence was highest among patients who were ALK-positive (31.6%) or had EGFR-positive (29.4%). The incidence rates were observed to be 0.086 in the EGFR group (95% CI 0.045–0.131), 0.062 in the ALK group (95% CI 0.003–0.122), 0.057 in the KRAS group (95% CI 0.000–0.188), 0.064 in the ROS1 group (95% CI 0.000–0.162), and 0.055 in the RET group (95% CI 0.000–0.224).

**Interpretation:**

Comprehensive meta-analyses indicate prevalence and incidence of BM are higher in patients with specific genomic alterations and advanced disease. Brain imaging and targeted therapies for brain penetrance are important.

**Supplementary Information:**

The online version contains supplementary material available at 10.1007/s12672-025-03621-w.

## Introduction

Lung cancer is the second most prevalent malignancy globally and the leading cause of cancer-related mortality [1]. It is classified into two primary types: NSCLC and small cell lung cancer (SCLC), with NSCLC representing approximately 85% of all cases [2, 3]. At diagnosis, 20% of patients present with BM, and as the disease advances, the incidence of BM in NSCLC cases rises to 50% [4, 5]. Furthermore, the survival time for patients with BM averages only about 5 months, posing a significant threat to patient lives [6]. Historically, treatment strategies were primarily guided by disease stage or histological classification, distinguishing between squamous and non-squamous types. In contrast, contemporary approaches emphasize the significance of actionable genomic alterations, including EGFR, ALK, ROS1, and other less prevalent mutations, which have emerged as critical factors in determining optimal treatment options. Survival rates for BM from NSCLC are improving due to a combination of novel targeted agents against actionable genomic alterations, early diagnosis, radiotherapy, and rapid advances in therapeutic approaches such as immunotherapy [7]. Studies have demonstrated that targeted therapy for tumors possessing specific genomic alterations, such as EGFR and ALK, can significantly enhance the overall survival rate of patients with BM [8–10]. Consequently, we have reason to believe that targeted therapies designed for specific genomic alterations may serve as a significant approach for the treatment of NSCLC and its BM in the future.

Consequently, systematic reviews and meta-analyses addressing the incidence and prevalence of BM in NSCLC, particularly those stratified by genomic alterations, hold significant value. In 2023, Gillespie et al. [11] published a meta-analysis of 64 studies that revealed the highest prevalence and incidence of BM in patients with ALK-positive (34.9%) and RET translocation (32.2%) alterations. These findings have significant implications for targeting patients with specific genomic alterations and for enhancing patient survival. However, this study exclusively focused on patients with advanced (stage III) and metastatic (stage IV) NSCLC, excluding those with stage I and II disease. The inclusion of these early patients is significant, as they typically exhibit longer survival periods, thereby enhancing the investigation into the prevalence and incidence of BM. To address this shortcoming, we conducted a meta-analysis of all genomically altered NSCLC patients from 2000 to 2024. Subgroup analysis of patients was conducted to present the results of varying genomic brain metastasis burdens in both early-stage and late-stage patients. This can enhance the understanding of the risk of brain metastasis associated with specific genomic alterations for both doctors and patients, thereby offering theoretical support for personalized treatment and monitoring strategies in high-risk patient subgroups.

## Material and methods

### Search strategy and selection criteria

We adhered to the PRISMA guidelines while conducting this meta-analysis, and we registered it with PROSPERO (CRD42022315915) [12]. We conducted a search of the MEDLINE, EMBASE, and Cochrane systematic review databases for full-text articles published in English between January 1, 2000, and February 28, 2024. The search terms included a combination of “lung”, “met”, and “incidence” (Supplementary Tables 1–3), and the population, intervention, comparator, outcome, and study design criteria was used (Supplementary Tables 4). We included studies involving adults (aged 16 years and older) with NSCLC that reported the prevalence, incidence, or both of BM at the time of diagnosis. We excluded conference abstract studies and those published prior to January 2000, as studies from the pre–MRI era significantly impact the detection of BM [13]. Additionally, we excluded studies involving selected populations, including those that focused exclusively on BM, as well as studies for which stage-specific data were not available.

Three authors independently screened titles, abstracts, and full text to include articles. Disagreement for a particular assessment was resolved by discussing the issues with the fourth partner (LB) until a consensus was reached.

### Data extraction

Data extraction was conducted thoroughly and in duplicate by a minimum of two authors for each article. The following data were collected for the included studies: the year of publication, the name of the journal, the type of study (randomized controlled trial or observational), and the stages of NSCLC represented (stage I, stage II, stage III, stage IV, or mixed). In cases where the study was an RCT, details regarding the intervention and type of treatment were documented, including examples such as tyrosine kinase inhibitors (TKIs), chemotherapy, and prophylactic cranial irradiation (PCI). But we excluded treatment arms that included PCI as an intervention, as PCI is not currently considered standard of care and may influence the incidence of BM.

The numerical data extracted from each study encompassed the following components: (1) the total population diagnosed with NSCLC; (2) the number of patients presenting with BM at the time of diagnosis; (3) the prevalence of BM at diagnosis; (4) the median follow-up duration in months; (5) the total number of patients without BM who underwent follow-up; (6) the total number of patients who subsequently developed BM; (7) the overall incidence of BM; and (8) the incidence rate of BM per year of patient follow-up.

### Quality assessment

Retrospective studies and randomized controlled trials were evaluated using the Newcastle Ottawa Scale [14]. The quality of all studies was independently assessed by the same two authors (YZ and LB). Any disagreements that arose were resolved through thorough discussion until a consensus was reached.

### Definitions

A study is classified as a 'mixed' cohort if it includes patients from more than two clinical trial phases (Stage I, II, III, or IV) simultaneously. Annual incidence rates were calculated by dividing the number of patients who developed BM during the follow-up period by the number of patients without BM at the beginning of the study. This proportion was subsequently divided by the length of the follow-up period in months and then multiplied by 12 to yield an annual rate.

### Statistical analysis

In the ALK-positive subgroup, we analyzed data for all patients and subsequently excluded those treated with second- and third-generation TKIs, such as alectinib, brigatinib, and lorlatinib, which are known to reduce the incidence of BM [15, 16]. In the meta-analysis, we employed random-effects models to generate pooled proportion estimates for prevalence at diagnosis, and we conducted single-incidence meta-analyses to assess annual incidence. We generated forest plots to illustrate incidence based on a random intercept generalized linear mixed model. For each random effects model, we assessed heterogeneity utilizing the maximum restricted likelihood estimator [17, 18]. Prevalence was computed using pooled proportions methods, specifically employing the inverse variance method. We calculated total heterogeneity and *I*^*2*^ statistics. Additionally, publication bias was evaluated and presented through funnel plots. This meta-analysis was conducted using the “meta” package in R statistical software version 4.2.1.

## Results

### Systematic review and characteristics

**Fig. 1:**
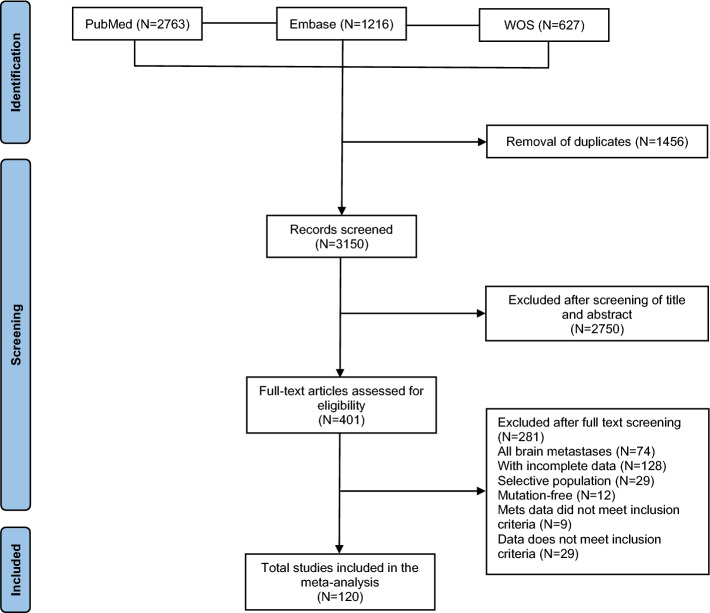
PRISMA flow diagram, of study selection for inclusion in this review and meta-analysis

After a comprehensive review, 401 studies were identified as potentially suitable for inclusion. Subsequently, 281 studies were excluded (Supplementary Table 5), leading to a final total of 120 studies that fulfilled the specified inclusion and exclusion criteria for analysis (Fig. 1). Among these, 120 studies provided prevalence data, while 85 studies reported incidence data and 94 of these studies both provided prevalence data and reported incidence data.

### Baseline characteristics

This study encompassed a total of 120 studies, with their baseline characteristics detailed in Supplementary Table 6–7. The median number of patients included per study was 160.9, with an interquartile range (IQR) of 46 to 192 and an overall range from 3 to 1189. Among these studies, 94 reported the brain metastatic status at diagnosis, encompassing a total of 17,542 patients. Meanwhile, 85 studies provided data on the incidence of BM in patients who did not initially present with these metastases, involving 12,425 patients. The studies reviewed employed various methodologies to assess genomic alterations, including fluorescence in situ hybridization, next-generation sequencing, array-based techniques, and combinations of these methods.

### Prevalence of BM at diagnosis

A total of 94 studies, encompassing 17,485 patients diagnosed with advanced NSCLC without BM, reported pooled prevalence rates. The pooled prevalence was 28.8% (95% CI 0.263–0.313) (Table 1). The pooled prevalence among stage I-II studies was 19.8% (95% CI 0.031–0.365). The pooled prevalence among stage III-IV studies was 26.3% (95% CI 0.228–0.298). The pooled prevalence among stage IV studies was 30.3% (95% CI 0.269–0.337). The pooled prevalence among stage mixed (stage I or II or III or IV) studies was 28.6% (95% CI 0.232–0.341).

**Table 1:** Prevalence and incidence of brain metastases in NSCLC, stratified by genomic

Category	Included studies (number of patients)	Median follow-up in year (IQR)	Number developing BM (%)	Pooled incidence of incidence per y (95% CI)
All studies Stage I-II	95 (13,323) 2 (53)	NA	3909 (29.3) 23 (43.4)	0.035 [0.000; 0.114]
Stage IV	14 (1556)	2.10 [1.13–3.20]	544 (35.0)	0.127 [0.005; 0.249]
Stage III	2 (92)	2.56 [1.87–3.27]	37 (40.2)	0.146 [0.000; 0.457]
Stage III − IV	48 (6534)	1.36 [1.64–3.00]	1788 (27.4)	0.087 [0.035; 0.138]
Stage mixed	33 (4029)	1.90 [1.73; 3.63]	1093 (27.1)	0.062 [0.013; 0.111]
Unstaged information	8 (1059)	4.33[1.25–5.58]	424 (40.0)	0.148 [0.006; 0.290]
EGFR	62 (9662)	1.79 [1.63; 3.42]	3001 (31.1)	0.088 [0.045; 0.131]
ALK	20 (1871)	1.96 [1.67; 3.63]	501 (26.8)	0.062 [0.003; 0.122]
ROS1	7 (212)	3.16 [1.84; 5.00]	63 (29.7)	0.064 [0.000; 0.162]
KRAS	5 (409)	1.71 [1.50–3.21]	100 (24.4)	0.057 [0.000; 0.188]
HER2	3 (207)	1.79 [1.48–2.49]	51 (24.6)	0.136 [0.000; 0.420]
RET	4 (216)	2.49 [1.72–4.21]	75 (34.7)	0.055 [0.000; 0.224]
MET	2 (72)	2.84 [1.79–3.46]	17 (23.6)	0.081 [0.000; 0.288]

Category	Included Studies (Number of Patients)	Number With BM (%)	Pooled Prevalence (95% CI)
Prevalence			
All studies	94 (17,485)	5149 (29.4)	0.288 [0.262; 0.313]
Stage I-II	2 (68)	15 (22.1)	0.198 [0.031; 0.365]
Stage IV	22 (2500)	760 (30.4)	0.303 [0.269; 0.337]
Stage III	2 (113)	28 (24.7)	0.141 [0.000; 0.415]
Stage III − IV	49 (8861)	2330 (26.3)	0.263 [0.228; 0.298]
Stage mixed	24 (2713)	779 (28.7)	0.286 [0.232; 0.341]
Unstaged information	11 (3230)	1108 (34.3)	0.410 [0.286; 0.534]
EGFR	60 (12,800)	3745 (29.3)	0.294 [0.260; 0.327]
ALK	22 (2378)	757 (31.8)	0.316 [0.259; 0.372]
ROS1	6 (241)	48 (19.9)	0.165 [0.049; 0.280]
KRAS	11 (1235)	303 (24.5)	0.243 [0.185; 0.301]
HER2	2 (142)	33 (23.2)	0.243 [0.124; 0.362]
BRAF	3 (287)	149 (51.9)	0.484 [0.000; 0.620]
MET	2 (86)	14 (16.2)	0.159 [0.081; 0.237]
RET	4 (316)	100 (31.6)	0.385 [0.227; 0.543]

### BM prevalence in patients with specific genomic alterations

Pooled prevalence forest plots are illustrated in Supplementary Fig. 1–6. The pooled prevalence in the EGFR positive group was 29.4%, based on 60 studies involving 12,800 patients (95% CI 0.260–0.327); the pooled prevalence in the EGFR positive group of stage III-IV was 27.8% (95% CI 0.241–0.316); the pooled prevalence in the EGFR positive group of stage mixed was 24.8% (95% CI 0.173–0.322). The pooled prevalence in the ALK positive group was 31.6%, based on 22 studies involving 2378 patients (95% CI 0.259–0.372); the pooled prevalence in the ALK positive group of stage III-IV was 26.7% (95% CI 0.187–0.348); the pooled prevalence in the ALK positive group of stage mixed was 35.4% (95% CI 0.313–0.39). The pooled prevalence in the KRAS positive group was 24.3%, based on 11 studies involving 1235 patients (95% CI 0.185–0.301). The pooled prevalence in the ROS1 positive group was 16.5%, based on 6 studies involving 241 patients (95% CI 0.049–0.280). The pooled prevalence in the RET positive group was 38.5%, based on 4 studies involving 316 patients (95% CI 0.227–0.543). The pooled prevalence in the HER2 positive group was 24.3%, based on 2 studies involving 142 patients (95% CI 0.124–0.362). The pooled prevalence in the MET positive group was 15.9%, based on 2 studies involving 86 patients (95% CI 0.081–0.237).

### Prevalence among other genomic subtypes: BRAF, PI3K, FGFR1, ERBB2

Due to the inclusion of certain genomic subtypes in fewer than two studies, the results can only be presented descriptively (Supplementary Table 8). The analysis encompassed three groups of genotypes: BRAF, PI3K, FGFR1, and ERBB2, which exhibited prevalence rates of 50%, 27.3%, 0%, and 0%, respectively.

### Pooled incidence of BM

A total of 95 studies, encompassing 13,323 patients diagnosed with advanced NSCLC without BM, reported pooled incidence rates. The pooled incidence per year was 0.076 (95% CI 0.045–0.106) (Table 1). The pooled incidence per year among stage I-II studies was 0.035 (95% CI 0.000–0.114). The pooled incidence per year among stage III studies was 0.146 (95% CI 0.000–0.457). The pooled incidence per year among stage IV studies was 0.127 (95% CI 0.005–0.249). The pooled incidence per year among stage III-IV studies was 0.087 (95% CI 0.035–0.138). The pooled incidence per year among mixed (stage I or II or III or IV) studies was 0.062 (95% CI 0.013–0.111).

### Pooled incidence stratified by genomic alterations

**Fig. 2:**
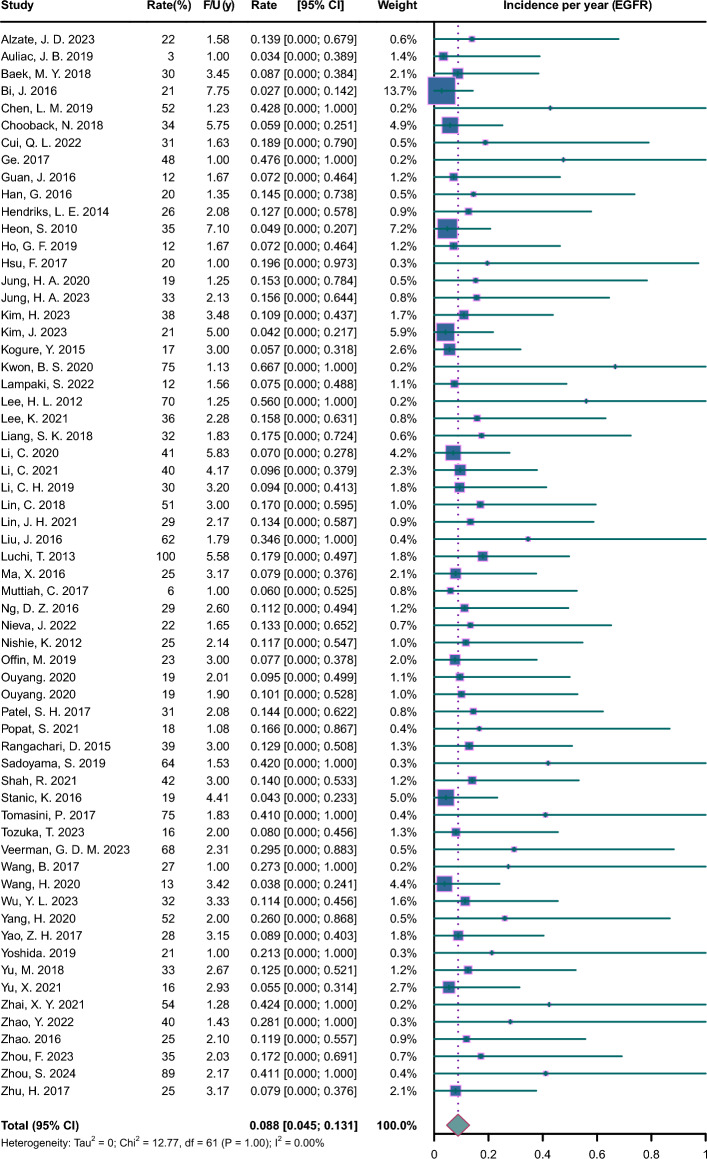
Forest plot of incidence per year in EGFR-positive NSCLC. CI, confidence interval; F/U, follow-up

**Fig. 3:**
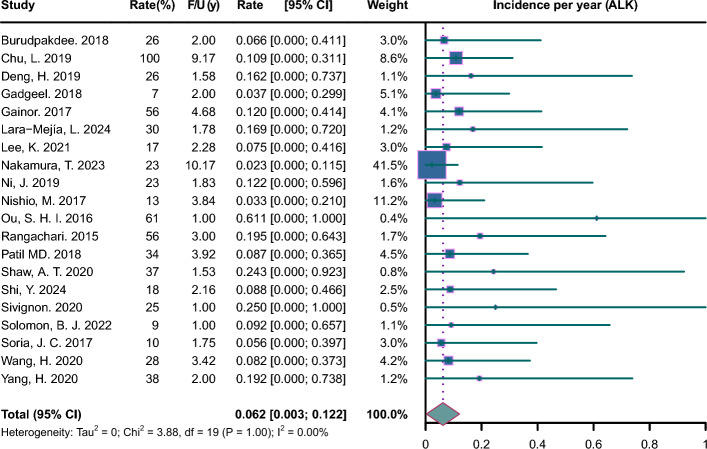
Forest plot of incidence per year in ALK-positive NSCLC. CI, confidence interval; F/U, follow-up

**Fig. 4:**
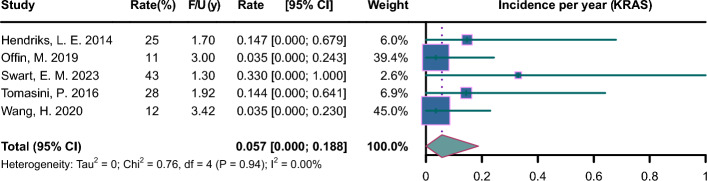
Forest plot of incidence per year in KRAS-positive NSCLC. CI, confidence interval; F/U, follow-up

**Fig. 5:**
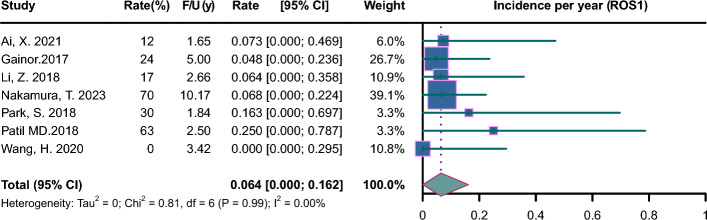
Forest plot of incidence per year in ROS1-positive NSCLC. CI, confidence interval; F/U, follow-up

**Fig. 6:**
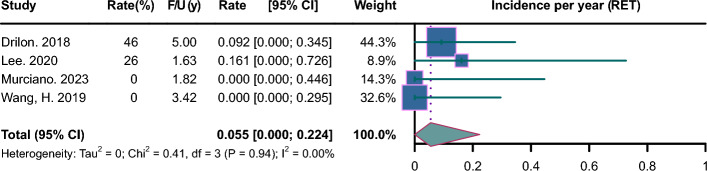
Forest plot of incidence per year in MET-positive NSCLC. CI, confidence interval; F/U, follow-up

**Fig. 7:**
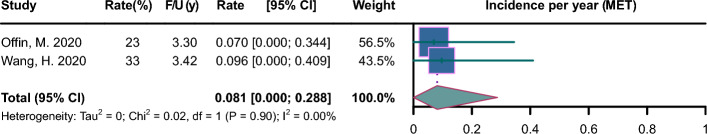
Forest plot of incidence per year in RET-positive NSCLC. CI, confidence interval; F/U, follow-up

Pooled incidence plots are illustrated in Figs. 2, 3, 4, 5, 6, 7. The pooled incidence of new BM was 0.088 per year in the EGFR-positive group, based on 62 studies involving 9662 patients (95% CI 0.045–0.131); the pooled incidence of new BM was 0.085 per year in the EGFR-positive group of stage III-IV (95% CI 0.023–0.147); the pooled incidence of new BM was 0.069 per year in the EGFR-positive group of stage mixed (95% CI 0.000–0.137). The pooled incidence of new BM was 0.062 per year in the ALK-positive group, based on 20 studies involving 1871 patients (95% CI 0.003–0.122);); the pooled incidence of new BM was 0.094 per year in the ALK-positive group of stage III-IV (95% CI 0.000–0.218); the pooled incidence of new BM was 0.068 per year in the ALK-positive group of stage mixed (95% CI 0.000–0.188). The pooled incidence of new BM was 0.057 per year in the KRAS-positive group, based on 5 studies involving 409 patients (95% CI 0.000–0.188). The pooled incidence of new BM was 0.064 per year in the ROS1-positive group, based on 7 studies involving 212 patients (95% CI 0.000–0.162). The pooled incidence of new BM was 0.055 per year in the RET-positive group, based on 4 studies involving 216 patients (95% CI 0.000–0.224). The pooled incidence of new BM was 0.136 per year in the HER2-positive group, based on 3 studies involving 207 patients (95% CI 0.000–0.420). The pooled incidence of new BM was 0.081 per year in the MET-positive group, based on 2 studies involving 72 patients (95% CI 0.000–0.288).

### Incidence among other genomic subtypes: BRAF, ERCC1, ERBB2

Due to the inclusion of certain genomic subtypes in fewer than two studies, the results can only be presented descriptively (Supplementary Table 8). The analysis encompassed three groups of genotypes: BRAF, ERCC1, ERBB2. Notably, the BRAF -positive group exhibited the highest annual incidence rate at 0.15, while the annual incidence of ERCC1 and ERBB2 positive groups was 0.03 and 0.04, respectively.

### Combined prevalence and incidence at the end of follow-up period

**Fig. 8:**
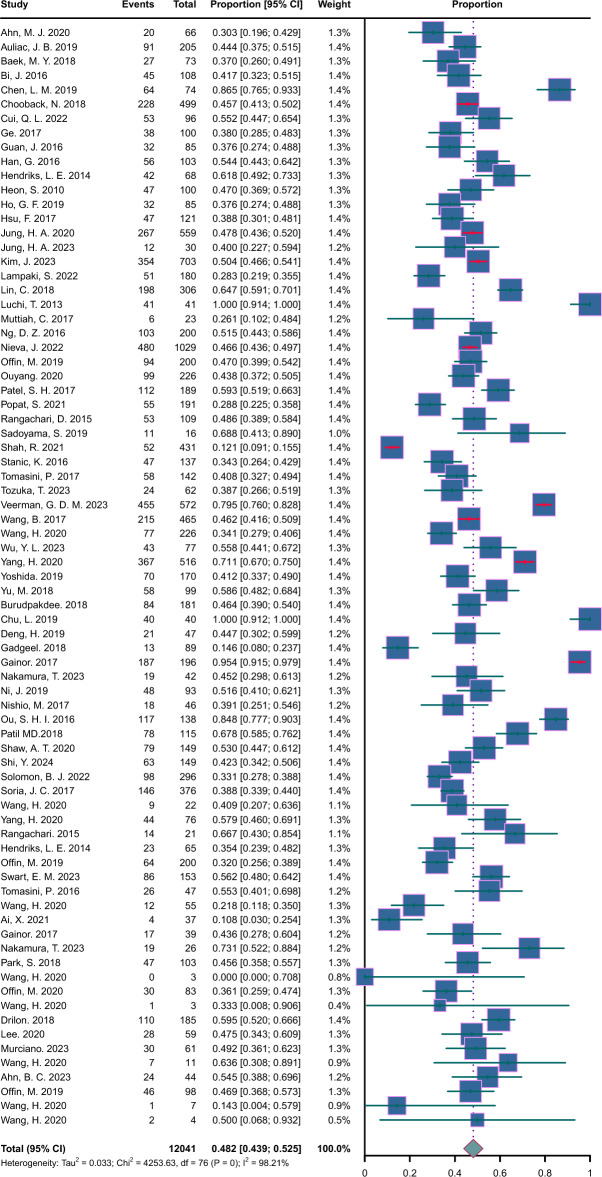
Forest plot of combined Prevalence and Incidence in NSCLC. CI, confidence interval; F/U, follow-up

A total of 64 studies involving 12,041 patients reported both the prevalence and the number of patients who developed BM by the end of the follow-up period (Fig. 8). Among these studies, the combined incidence and prevalence at the conclusion of the study period—median duration of 2.8 years—was found to be 48.2% (IQR: 0.431–0.517); the combined incidence and prevalence rate of EGFR positive group was 48.1% (IQR: 0.428–0.534); the combined incidence and prevalence rate of ALK positive group was 54.5% (IQR: 0.434–0.656); the combined incidence and prevalence rate of KARS positive group was 40.0% (IQR: 0.268–0.531); the combined incidence and prevalence rate of ROS1 positive group was 35.9% (IQR: 0.113–0.605).

###  Assessment of bias

The assessment of bias for all studies, utilizing the Newcastle–Ottawa Scale, is presented in Supplementary Fig. 3. The mean score across all studies was 6.3 out of a maximum total of 9. The funnel plots for each forest plot generated are illustrated in Supplementary Fig. 8.

## Discussion

This study represents the first systematic review and meta-analysis that integrates both prevalence and incidence data of BM across progression cycles in metastatic NSCLC with targeted genomic alterations. In our exploration of prevalence, we included a total of 94 studies encompassing 17,542 patients. The initial diagnosis indicated a pooled prevalence of 28.8%. The results revealed that the prevalence of targeted genomic alterations was as follows: EGFR (29.4%), ALK (31.6%), KRAS (24.3%), ROS1 (16.5%), RET (38.5%), HER2 (24.3%), and MET (15.9%). In our exploration of prevalence, we included a total of 85 studies encompassing 12,425 patients. The initial diagnosis indicated a pooled incidence per year was 0.076. The results revealed that the pooled incidence per year of targeted genomic alterations was as follows: EGFR (0.088), ALK (0.062), KRAS (0.057), ROS1 (0.064), RET (0.055), HER2 (0.136), and MET (0.081). Regardless of prevalence or incidence, the occurrence of BM in the EGFR-positive group was significantly higher than the average. The EGFR gene mutation is the most common mutation among Asian patients with NSCLC and the second most common mutation in Caucasian patients with the same condition [19, 20]. Therefore, it is recommended that patients with EGFR gene mutations undergo regular testing to mitigate the risk of brain metastasis.

The significantly elevated prevalence of BM observed in specific genomic subgroups, particularly in RET fusion-positive NSCLC (38.5%), highlights the presence of distinct biological mechanisms that confer neurotropic potential. RET fusions lead to constitutive kinase activation, primarily signaling through the RAS/MAPK and PI3K/AKT pathways [21]. This aberrant signaling orchestrates a pro-metastatic cascade that facilitates brain colonization. Key mechanisms include the upregulation of matrix metalloproteinases (MMPs), particularly MMP-9. MMP-9 plays a critical role in degrading extracellular matrix components, notably type IV collagen, which is a major constituent of the basement membrane of the blood–brain barrier (BBB). This enzymatic degradation disrupts the integrity of the BBB, enabling tumor cell extravasation into the brain parenchyma [22, 23]. The high prevalence at diagnosis indicates a tendency for RET-driven tumors to infiltrate the brain early in the disease progression. In contrast, the relatively lower annual incidence rate (0.055) observed in our RET cohort may suggest the presence of dormant micrometastases following this initial infiltration, with subsequent clinical manifestations reliant on reactivation within the brain microenvironment [24].

In addition to RET fusion, we also found that EGFR mutations have a high prevalence and incidence associated with ALK rearrangements. Although both EGFR mutations and ALK rearrangements can drive brain metastasis, their mechanisms of action differ fundamentally. EGFR mutations primarily rely on direct intervention from the microenvironment: they upregulate MMPs (such as MMP-9) to degrade components of the blood–brain barrier [25], compromising its integrity, while simultaneously secreting chemokines to mediate the directional migration of tumor cells towards brain tissue. Additionally, the high expression of IL-11 acts as a ligand for EGFR/gp130, activating downstream signals that increase PD-L1 expression, thereby inducing immune evasion to sustain the survival of brain metastatic lesions [26]. The ALK rearrangement focuses on intracellular programmed regulation: the activation of the IGF1/IGF1R pathways drives migration through the RAS-RAC1-MAPK/ERK pathway, while the PI3K-AKT-mTOR-p70S6K pathway promotes proliferation and adaptation to the microenvironment [27–29]. MTOR enhances invasiveness by inducing HIF-1α and E-Syt1, ultimately relieving the suppression of EMT transcription factors through the ZEB1/miR-200c axis[30], triggering epithelial-mesenchymal transition and systematically endowing distant colonization capabilities. The EGFR primarily centers around microenvironment disruption and immune evasion, while ALK is dominated by the integration of intracellular dual pathways and the EMT program. Therefore, EGFR should focus on penetrating the blood–brain barrier in combination with immune modulators, whereas ALK needs to block the synergistic effects of EMT and the dual pathways.

The prevalence of BM in patients at stages I − II was 0.198 [0.031; 0.365], while in patients at stages III − IV it was 0.263 [0.228; 0.298]. In patients with mixed-stage cancer, the prevalence was 0.286 [0.232; 0.341]. The prevalence of mixed-stage patients is higher than that of stage III-IV patients. We posit that the majority of the mixed-stage patients included in this study are, in fact, late-stage patients, with little to no data available on early-stage patients. Regarding this aspect of the study, we anticipate that future researchers will incorporate additional data to achieve more comprehensive results. These findings indicate that the prevalence of BM is significantly lower in patients with early-stage NSCLC compared to those in later stages. Moreover, most patients in the early stages typically exhibit no obvious symptoms; symptoms tend to manifest only when tumors have reached a certain size, which corresponds to the middle or late stages of the disease [31–33]. Therefore, enhancing the screening processes for NSCLC is of paramount importance. Our results suggest that for high-risk groups, such as middle-aged and elderly individuals, regular tumor screening could significantly reduce the prevalence of BM in patients with advanced NSCLC. The annual incidence rate of brain conditions was 0.035 [0.000–0.114] for patients with stage I-II, and 0.146 [0.000–0.457] for patients with stage III-IV. For patients in the mixed phase, the annual incidence rate was 0.062 [0.013–0.111]. These findings indicate that the annual incidence rate for early-stage patients is significantly lower than that for late-stage patients, highlighting the importance of selecting appropriate treatment options for those in the later stages.

Research indicates that BM frequently manifest either at the initial diagnosis of NSCLC or during the progression of the disease. Approximately 10% of newly diagnosed NSCLC patients present with BM at the outset, while the incidence rises to about 30% among patients with advanced NSCLC [34–36]. There are also studies showing that the population prevalence at the time of diagnosis is estimated to be between 20 and 60% [37, 38]. Our study indicates that the prevalence of patients with NSCLC is approximately 30%, while the prevalence of early-stage patients is around 20%. This finding helps to explore the true incidence of BM. The differences in the incidence of BM reported across various studies may be attributed to the use of differing diagnostic methods. Conor et al. suggest that cranial imaging examinations should be included during baseline staging, particularly in cases of metastatic lung cancer. Given that the diagnosis often reveals a high prevalence—up to 60%—this approach can help alleviate the pain and economic burden associated with BM from NSCLC [11, 39–42]. The pooled incidence per year of patients with stage III and stage IV NSCLC was 0.146 and 0.127, respectively, significantly exceeding that of patients with stages I and II. Consequently, the burden of BM in the natural progression of advanced and metastatic NSCLC is substantial. Future research should prioritize the prevention, management, and treatment of BM.

## Limitations

This meta-analysis has several limitations that should be acknowledged. First, there are limitations related to language; we only included articles published in English, which may lead to the omission of relevant data published in other languages. This could introduce selection bias and limit the comprehensiveness of our research findings. Secondly, regarding the shortcomings in addressing heterogeneity, to maximize data inclusion and obtain pooled estimates of prevalence and incidence, we combined studies involving patients treated with various kinase inhibitors and, in some instances, immunotherapy. While the impact of these treatments on outcomes is minimal, they represent a confounding factor that cannot be overlooked. Thirdly, there is an issue regarding the calculation of follow-up time. We utilized the median follow-up time rather than the mean follow-up time to calculate the annual incidence rate of BM. Given that survival times often exhibit bias, this approach may lead to an underestimation of the time patients are at risk and an overestimation of the annual incidence rate of BM.

Fourth, there is a lack of adequate research design and data availability, as most of the included studies are retrospective in nature. Furthermore, due to the absence of critical data, such as median follow-up time or specific disease staging information, these relevant studies were excluded, which reduced the overall sample size and may introduce bias. Fifth, there is a lack of data on early-stage patients and rare genomic subtypes. Despite our extensive search, data on early (I-II) NSCLC patients remain relatively scarce, which limits the accuracy of prevalence and incidence estimates for this subgroup. Furthermore, studies and patient numbers concerning several genomic subtypes, such as RET, HER2, MET, BRAF, ERCC1, ERBB2, PI3K, and FGFR1, are also minimal (< 5 studies), resulting in wider confidence intervals for these specific alterations and lower reliability of the aggregated estimates. Finally, the included studies exhibited considerable heterogeneity and publication bias. Although our goal was to incorporate as many studies as possible to accurately determine the natural history and incidence of brain metastasis and to mitigate between-study heterogeneity through the use of random effects models, these factors may still affect the accuracy of our results.

## Conclusions

This comprehensive meta-analysis, encompassing 120 studies across all stages of NSCLC, quantifies the risks of BM specific to driver genes. RET fusion demonstrates the highest prevalence at diagnosis, recorded at 38.5%, whereas tumors with HER2 alterations exhibit the most significant annual incidence of BM at 0.136. Elevated burdens of BM are observed in subgroups with EGFR mutations (prevalence: 29.4%; incidence: 0.088) and ALK rearrangements (prevalence: 31.6%). Notably, advanced-stage (III–IV) disease correlates with a 2.7-fold increase in annual BM incidence compared to early stages (0.087 vs. 0.035). These findings underscore that genomic stratification surpasses traditional staging in predicting BM risk, identifying RET, HER2, EGFR, and ALK as high-priority targets for enhanced CNS surveillance. We recommend baseline brain MRI and regular monitoring for these subgroups, in conjunction with CNS-penetrant tyrosine kinase inhibitors. Future research should address existing data gaps in early-stage NSCLC and rare genomic variants to optimize personalized preventive strategies.

## Data sharing

Because this meta-analysis was based on data extracted from previously published research, most of the data and study materials are available in the public domain. For further discussions, we invite interested parties to contact the corresponding author.

## Supplementary Information

Additional file 1 (PDF 2207 KB)

## Data Availability

Some or all data, models, or code generated or used during the study are available from the corresponding author by request.
