# Supplementary material for: Driver gene-specific prevalence and incidence of brain metastases in non-small cell lung cancer: a meta-analysis encompassing all disease stages
Source: Discov Oncol. 2025 Sep 30;16:1780. doi: 10.1007/s12672-025-03621-w (PMC12484510; doi:10.1007/s12672-025-03621-w)

Supplementary Table 1. PubMed search strategy (Search performed February 2024).

Search	Query	Results
1	(lung cancer) or (lung carcinoma) or (non-small cell lung cancer) or (NSCLC) or (lung neoplasm*)	335590
2	(Brain metasta*) OR (CNS metasta*) OR (central nervous system metasta*)	36576
3	(incidence) OR (Incidence)	3034433
4	1 and 2 and 3	2923
5	4 not (animals not humans)	2914
6	Limit 5 to English	2763

Supplementary Table 2. Embase search strategy (Search performed February 2024).

Search	Query	Results
1	(lung cancer) or (lung carcinoma) or (non-small cell lung cancer) or (NSCLC) or (lung neoplasm*)	136687
2	(Brain metasta*) OR (CNS metasta*) OR (central nervous system metasta*)	1687657
3	(incidence) OR (Incidence)	1312376
4	1 and 2 and 3	1278
5	4 not (animals not humans)	1266
6	Limit 5 to English	1216

Supplementary Table 3. Web Of Science search strategy (Search performed February 2024).

Search	Query	Results
1	(lung cancer) or (lung carcinoma) or (non-small cell lung cancer) or (NSCLC) or (lung neoplasm*)	199142
2	(Brain metasta*) OR (CNS metasta*) OR (central nervous system metasta*)	19378
3	(incidence) OR (Incidence)	307263
4	1 and 2 and 3	634
5	4 not (animals not humans)	630
6	Limit 5 to English	627

Supplementary Table 4. PICOS inclusion criteria

**Review Question**	In patients with non-small cell lung cancer (NSCLC), what is the prevalence of brain metastases (BM) at the time of diagnosis and what is the annual incidence? Additionally, does this vary based on the presence of known genomic alterations?	
**Population**	Adults ⩾ 16 years with advanced or metastatic NSCLC.<br>This review excludes studies published prior to January 2000, representative populations of non-small cell lung cancer cohorts, and patients who received prophylactic cranial irradiation.	
**Intervention**	Immunotherapy, Chemotherapy, BM Screening programmes	
**Comparator**	Stage, Genomic alterations	
**Outcomes**	Incidence per year	Prevalence stratified by stage
	Genomic alteration specific incidence	Incidence stratified by screening programme use
	Overall prevalence at diagnosis	Number of studies with genomic alteration data available
**Setting**	Studies taking place in any hospital.	
**Study design**	Prospective case series and cohort studies with >1 adult patient	
**Follow-up**	Report the incidence of BM at diagnosis, annual incidence, or median follow-up time.	

Supplementary Table 5. List of full-text articles screened and excluded, including reasons for exclusion (N=281).

Author, year	Title	Reason for exclusion
**An, N. 2019**	Therapeutic effect of first-line EGFR-TKIs combined with concurrent cranial radiotherapy on NSCLC patients with EGFR activating mutation and brain metastasis: A retrospective study	Selective population-only included BM patients
**Armocida, D. 2023**	EGFR-Driven Mutation in Non-Small-Cell Lung Cancer (NSCLC) Influences the Features and Outcome of Brain Metastases	Selective population- only included BM patients
**Armour, A. 2007**	Gefitinib in advanced non-small cell lung cancer: Clinical experience in patients of Asian origin	Data does not meet inclusion criteria (No patient-specific data were included)
**Arrieta, O. 2018**	Prophylactic Cranial Irradiation Reduces the Risk of Brain Metastases in High-Risk Lung Cancer Patients: EGFR and ALK Mutations	Selective population- PCI
**Arrieta, O. 2020**	Response rate of patients with baseline brain metastases from recently diagnosed non-small cell lung cancer receiving radiotherapy according to EGFR, ALK and KRAS mutation status	Selective population- only included BM patients
**Babu Koyyala, V. P. 2018**	Frequency of T790M mutations after progression on epidermal growth factor receptor tyrosine kinase inhibitor in metastatic non-small cell lung cancer in Indian patients: real-time data from tertiary cancer hospital	Selective population- after treatment with TKI
**Balasubramanian, S. K. 2020**	Impact of EGFR mutation and ALK rearrangement on the outcomes of non-small cell lung cancer patients with brain metastasis	Selective population- only included BM patients
**Bauer, T. M. 2020**	Brain Penetration of Lorlatinib: Cumulative Incidences of CNS and Non-CNS Progression with Lorlatinib in Patients with Previously Treated ALK-Positive Non-Small-Cell Lung Cancer	With incomplete data
**Bearz, A. 2010**	Activity of Pemetrexed on brain metastases from Non-Small Cell Lung Cancer	Selective population- only included BM patients

**Braganca, K. C.2010**	Efficacy and safety of bevacizumab in active brain metastases from non-small cell lung cancer	Selective population- only included BM patients
**Breindel, J. L. 2013**	EGF receptor activates MET through MAPK to enhance non-small cell lung carcinoma invasion and brain etastasis	Mets data does not meet inclusion criteria
**Burudpakdee, C. 2018**	Economic impact of preventing brain metastases with alectinib in ALK-positive non-small cell lung cancer	Data does not meet inclusion criteria
**Byeon, S. 2016**	Analysis of the benefit of sequential cranial radiotherapy in patients with EGFR mutant non-small cell lung cancer and brain metastasis	Selective population- only included BM patients
**Caballero, J. A. 2012**	Prognostic factors for survival in patients treated with stereotactic radiosurgery for recurrent brain metastases after prior whole brain radiotherapy	Selective population- only included BM patients
**Cai, L. 2014**	A comparative analysis of EGFR mutation status in association with the efficacy of TKI in combination with WBRT/SRS/surgery plus chemotherapy in brain metastasis from non-small cell lung cancer	Selective population- only included BM patients
**Calles, A. 2015**	Immunohistochemical Loss of LKB1 Is a Biomarker for More Aggressive Biology in KRAS-Mutant Lung Adenocarcinoma	Data does not meet inclusion criteria (No Specific data on brain metastasis)
**Chen, Y. 2021**	Analysis of metastases in non-small cell lung cancer patients with epidermal growth factor receptor mutation	Data for BMS patients before and after treatment are not clear, only total data
**Chen, Y. 2019**	Combination therapy of brain radiotherapy and EGFR-TKIs is more effective than TKIs alone for EGFR-mutant lung adenocarcinoma patients with asymptomatic brain metastasis	Selective population-only included BM patients
**Chen, Y. 2016**	First-line epidermal growth factor receptor (EGFR)-tyrosine kinase inhibitor alone or with whole-brain radiotherapy for brain metastases in patients with EGFR-mutated lung adenocarcinoma	Selective population- only included BM patients
**Cheng, W. C. 2020**	The Impact of Acquired EGFR T790M Mutation and EGFR Circulating Cell-Free DNA on Survival in	Mets data does not meet inclusion criteria

	Patients with Lung Adenocarcinoma Following EGFR-TKI Therapy	
**Choi, J. H. 2022**	The efficacy of EGFR-tyrosine kinase inhibitor in non-small cell lung cancer patients with synchronous brain metastasis: a real-world study	Selective population- only included BM patients
**Chow, L. Q. M.2022**	ASCEND-7: Efficacy and Safety of Ceritinib Treatment in Patients with ALK-Positive Non-Small Cell Lung Cancer Metastatic to the Brain and/or Leptomeninges	Data does not meet inclusion criteria
**Congedo, M. T.2012**	Surgery for oligometastatic non-small cell lung cancer: long-term results from a single center experience	With incomplete data
**Dasgupta, A. 2020**	Focal Leptomeningeal Disease with Perivascular Invasion in EGFR-Mutant Non-Small-Cell Lung Cancer	Data does not meet inclusion criteria（case analysis)
**De Carlo, E. 2018**	Outcomes of ALK positive lung cancer patients treated with crizotinib or second-generation ALK inhibitor: a monoinstitutional experience	With incomplete data
**Deng, C. 2021**	Prognostic value of epidermal growth factor receptor gene mutation in resected lung adenocarcinoma	With incomplete data
**Digumarthy, S. R. 2020**	Imaging Features and Patterns of Metastasis in Non-Small Cell Lung Cancer with RET Rearrangements	Data does not meet inclusion criteria
**Dinglin, X. X. 2013**	Pemetrexed and cisplatin combination with concurrent whole brain radiotherapy in patients with brain metastases of lung adenocarcinoma: a single-arm phase II clinical trial	With incomplete data
**Doherty, M. K. 2015**	Treatment of EGFR/ALK-driven non-small cell lung cancer (nsclc) brain metastases: Impact of first-line whole brain radiotherapy on outcome	Data does not meet inclusion criteria
**Drilon, A. 2018**	Frequency of Brain Metastases and Multikinase Inhibitor Outcomes in Patients With RET-Rearranged Lung Cancers	Data does not meet inclusion criteria
**Driscoll, J. 2013**	The brain microenvironment negatively regulates miRNA-768-3pin lung cancer to induce KRAS and promote metastasis	Data does not meet inclusion criteria

**Dubé-Pelletier, M. 2021**	Routine Neuroimaging in Patients with Stage IV Non-Small Cell Lung Cancer: A Single Center Experience	Data does not meet inclusion criteria (Not all included patients have genetic mutations
**Eichholz, J. 2023**	The Impact of Co-Alterations on Outcomes after Local Therapy for Patients with KRAS-Mutant Lung Adenocarcinoma Brain Metastases	Selective population- only included BM patients
**Farris, J. C. 2023**	Brain Metastasis Incidence and Patterns of Presentation After Definitive Treatment of Locally Advanced Non-Small Cell Lung Cancer: A Potential Argument for Brain Magnetic Resonance Imaging Surveillance	With incomplete data
**Franco, F. 2021**	Epidemiology, treatment, and survival in small cell lung cancer in Spain: Data from the Thoracic Tumor Registry	Data does not meet inclusion criteria (SCLC)
**Gadgeel, S. 2018**	Cumulative incidence rates for CNS and non-CNS progression in two phase II studies of alectinib in ALK-positive NSCLC	With incomplete data
**Gadgeel, S. M. 2014**	Safety and activity of alectinib against systemic disease and brain metastases in patients with crizotinib-resistant ALK-rearranged non-small-cell lung cancer (AF-002JG): results from the dose-finding portion of a phase 1/2 study	With incomplete data
**Gainor, J. F. 2017**	Patterns of metastatic spread and mechanisms of resistance to crizotinib in ROS1-positive non-small-cell lung cancer	With incomplete data
**Garon, E. B. 2013**	Preliminary clinical safety and activity of MK-3475 monotherapy for the treatment of previously treated patients with non-small cell lung cancer (NSCLC)	Data does not meet inclusion criteria (No Specific data on brain metastasis)
**Gaspar, L. E. 2005**	Time from treatment to subsequent diagnosis of brain metastases in stage III non-small-cell lung cancer: a retrospective review by the Southwest Oncology Group	Data does not meet inclusion criteria
**Gaye, E. 2019**	Intra-cranial efficacy of brigatinib in an ALK-positive non-small cell lung cancer patient presenting leptomeningeal carcinomatosis	Data does not meet inclusion criteria (only one patient case analysis)
**Ge, M. X. 2017**	High probability and frequency of EGFR mutations in non-small cell lung cancer with brain metastases	Selective population

**Gijtenbeek, R. G. P. 2020**	Nationwide Real-world Cohort Study of First-line Tyrosine Kinase Inhibitor Treatment in Epidermal Growth Factor Receptor-mutated Non-small-cell Lung Cancer	Data does not meet inclusion criteria (No Specific data on brain metastasis)
**Gu, Y. 2021**	Value and significance of brain radiation therapy during first-line EGFR-TKI treatment in lung adenocarcinoma with EGFR sensitive mutation and synchronous brain metastasis: Appropriate timing and technique	Selective population- only included BM patients
**Gui, Q. 2017**	Prolonged survival of patients with EGFR-mutated non-small cell lung cancer with solitary brain metastases treated with surgical resection of brain and lung lesions followed by EGFR TKIs	Selective population- only included BM patients
**Hansen, K. H. 2023**	Clinical outcomes of ALK+ non-small cell lung cancer in Denmark	With incomplete data
**Hendriks, L. E. L. 2019**	Survival of patients with non-small cell lung cancer having leptomeningeal metastases treated with immune checkpoint inhibitors	Data does not meet inclusion criteria (Out of 1288 patients, only 7 had genetic mutations)
**Herbst, R. S. 2023**	Adjuvant Osimertinib for Resected EGFR-Mutated Stage IB-IIIA Non Small-Cell Lung Cancer: Updated Results From the Phase III Randomized ADAURA Trial	Data does not meet inclusion criteria
**Horn, L. 2018**	Ensartinib (X-396) in ALK-Positive Non-Small Cell Lung Cancer: Results from a First-in-Human Phase I/II, Multicenter Study	With incomplete data
**Hsu, F. 2017**	Miliary metastases are associated with epidermal growth factor receptor mutations in non-small cell lung cancer: a population-based study	Unable to extract only EGFR mutation data
**Huang, Z. 2020**	Establishment of a Prognostic Nomogram for Lung Adenocarcinoma with Brain Metastases	Selective population- only included BM patients
**Hui, C. 2022**	Local control of brain metastases with osimertinib alone in patients with EGFR-mutant non-small cell lung cancer	Selective population- only included BM patients
**Huysentruyt, L. C. 2008**	Metastatic cancer cells with macrophage properties: Evidence from a new murine tumor model	Data does not meet inclusion criteria (not NSCLC)

**Imber, B. S. 2023**	Intracranial Outcomes of De Novo Brain Metastases Treated With Osimertinib Alone in Patients With Newly Diagnosed EGFR-Mutant NSCLC	Selective population- only included BM patients
**Isaksson, J. 2023**	KRAS G12C Mutant Non-Small Cell Lung Cancer Linked to Female Sex and High Risk of CNS Metastasis: Population-based Demographics and Survival Data From the National Swedish Lung Cancer Registry	Data does not meet inclusion criteria
**Isla, D. 2020**	Cost analysis of the management of brain metastases in patients with advanced ALK+ NSCLC: alectinib versus crizotinib	With incomplete data
**Ji, X. K. 2020**	The relationship between cerebrospinal fluid metastasis and gene mutations in non-small-cell lung cancer patients	Data does not meet inclusion criteria (Not all included patients have genetic mutations, selective people)
**Ji, Z. 2014**	Risk factors for brain metastases in locally advanced non-small cell lung cancer with definitive chest radiation	With incomplete data
**Jia, F. 2019**	Clinical research on stereotactic radiosurgery combined with epithermal growth factor tyrosine kinase inhibitors in the treatment of brain metastasis of non-small cell lung cancer	Data does not meet inclusion criteria (No Specific data on brain metastasis)
**Jiang, T. 2016**	EGFR TKIs plus WBRT Demonstrated No Survival Benefit Other Than That of TKIs Alone in Patients with NSCLC and EGFR Mutation and Brain Metastases	With incomplete data
**Jiang, Y. 2018**	Erlotinib versus gefitinib for brain metastases in Asian patients with exon 19 EGFR-mutant lung adenocarcinoma: a retrospective, multicenter study	Selective population- only included BM patients
**Jin, J. 2013**	Brain metastases as the first symptom of lung cancer: a clinical study from an Asian medical center	Selective population- only included BM patients
**Jin, Y. 2019**	Phosphorylated-Akt overexpression is associated with a higher risk of brain metastasis in patients with non-small cell lung cancer	With incomplete data
**Johung, K. L. 2013**	A clinical model for identifying radiosensitive tumor genotypes in non-small cell lung cancer	Selective population- only included BM patients

**Johung, K. L. 2016**	Extended Survival and Prognostic Factors for Patients With ALK-Rearranged Non-Small-Cell Lung Cancer and Brain Metastasis	With incomplete data
**Joshi, J. 2023**	An association of epidermal growth factor receptor mutation subtypes with prognostic prediction and site-specific recurrence in advanced stage lung cancer patients	Data does not meet inclusion criteria (No Specific data on brain metastasis)
**Jung, H. A. 2023**	Dacomitinib in EGFR-mutant non-small-cell lung cancer with brain metastasis: a single-arm, phase II study	Selective population- only included BM patients
**Kaler, A. K. 2023**	Mutational Analysis of EGFR Mutations in Non-Small Cell Lung Carcinoma-An Indian Perspective of 212 Patients	Data does not meet inclusion criteria
**Kang, L. 2023**	CNS efficacy of afatinib as first-line treatment in advanced non-small cell lung cancer patients with EGFR mutations	Selective population- only included BM patients
**Kim, K. 2023**	Clinical features and molecular genetics associated with brain metastasis in suspected early-stage non-small cell lung cancer	Data does not meet inclusion criteria (No treatment-related data available)
**Kim, M. 2021**	Development of Brain Metastases in Patients With Non-Small Cell Lung Cancer and No Brain Metastases at Initial Staging Evaluation: Cumulative Incidence and Risk Factor Analysis	data does not meet inclusion criteria (can't extract brain metastasis data for the two gene mutations separately)
**Krüger, S. 2011**	Brain metastasis in lung cancer. Comparison of cerebral MRI and 18F-FDG-PET/CT for diagnosis in the initial staging	With incomplete data
**Kudo, Y. 2015**	Prognostic Factors for Survival After Recurrence in Patients With Completely Resected Lung Adenocarcinoma: Important Roles of Epidermal Growth Factor Receptor Mutation Status and the Current Staging System	Data does not meet inclusion criteria
**Kuijpers, Cchj 2018**	Association of molecular status and metastatic organs at diagnosis in patients with stage IV non-squamous non-small cell lung cancer	Data does not meet inclusion criteria
**Kumar, M. 2020**	Discordance between Fluorescence In Situ Hybridization and Immunohistochemistry Analysis of	Data does not meet inclusion criteria (Not all

	Anaplastic Lymphoma Kinase Rearrangement in Indian Patients with Non-Small Cell Lung Cancer	included patients have genetic mutations
**Kumar, S. 2022**	Real-world Use of Radiation for Newly Diagnosed Brain Metastases in Patients With ALK-positive Lung Cancer Receiving First-line ALK Inhibitor	Selective population- only included BM patients
**Kwon, J. 2014**	Impact of multimodality approach for patients with leptomeningeal metastases from solid tumors	With incomplete data mutations
**Lara-Mejía, L. 2024**	Impact of Concurrent Genomic Alterations on Clinical Outcomes in Patients With ALK Rearranged NSCLC	With incomplete data
**Macerelli, M. 2013**	Does KRAS mutation status predicts for chemoresistance in advanced non-small cell lung cancer (NSCLC)?	With incomplete data
**Magnuson, W. J. 2016**	Impact of deferring radiation therapy in patients with epidermal growth factor receptor-mutant non-small cell lung cancer who develop brain metastases	With incomplete data
**Mahajan, A. 2018**	MR imaging radiomics of NSCLC brain metastases: A potential targetable imaging biomarker for EGFR status	With incomplete data
**Martín, C. 2018**	Real-World Treatment Patterns, Survival, and Prediction of CNS Progression in ALK-Positive Non-Small-Cell Lung Cancer Patients Treated with First-Line Crizotinib in Latin America Oncology Practices	With incomplete data
**Matsunaga, S. 2021**	Outcomes of Gamma Knife Radiosurgery for Brain Metastases From Anaplastic Lymphoma Kinase Rearrangement-Positive and EGFR Mutation-Positive Non-Small Cell Lung Cancer	All brain metastases
**Mills, M. N. 2022**	The presentation of brain metastases in melanoma, non-small cell lung cancer, and breast cancer and potential implications for screening brain MRIs	All brain metastases
**Miura, S. 2022**	Sequential Afatinib and Osimertinib in Asian Patients with EGFR Mutation-Positive Non-Small Cell Lung Cancer and Acquired T790M: Combined Analysis of Two Global Non-Interventional Studies	With incomplete data
**Mogenet, A. 2022**	Molecular profiling of non-small-cell lung cancer patients with or without brain metastases included in the randomized SAFIR02-LUNG trial and association with intracranial outcome	With incomplete data

**Moraes, F. Y. 2021**	Impact of EGFR mutation on outcomes following SRS for brain metastases in non-small cell lung cancer	With incomplete data
**Motta, M. 2011**	Gamma knife radiosurgery for treatment of cerebral metastases from non-small-cell lung cancer	With incomplete data<br>All brain metastases
**Mountzios, G. 2021**	An Observational Study to Assess the Molecular Epidemiology and Direct Medical Costs of Epidermal Growth Factor Receptor (EGFR) Mutations in Patients with Advanced EGFR Mutation-Positive Non-Small Cell Lung Cancer Treated with Afatinib in Real-World Clinical Settings in Greece	Mets data did not meet inclusion criteria
**Munarriz, B. J. 2019**	Mutational Landscape in Lung Cancer Patients by Targeted Next-Generation Sequencing and Differences by Gender in Spanish Population	With incomplete data
**Murase, K. 2017**	Clinical factors of long-term survival in patients with advanced lung cancer	Mets data did not meet inclusion criteria
**Na, Y. C. 2017**	Predictive factors of early distant brain failure after gamma knife radiosurgery alone in patients with brain metastases of non-small-cell lung cancer	All brain metastases
**Najjary, S. 2023**	Tumor lineage-specific immune response in brain metastatic disease: opportunities for targeted immunotherapy regimen?	All brain metastases
**Namba, Y. 2004**	Gefitinib in patients with brain metastases from non-small-cell lung cancer: review of 15 clinical cases	With incomplete data
**Ng, I. K. 2021**	Development, internal validation and calibration of a risk score to predict survival in patients with EGFR-mutant non-small cell lung cancer	Selective population - Recurrent or refractory lung cancer
**Ni, Y. 2016**	Local microwave ablation with continued EGFR tyrosine kinase inhibitor as a treatment strategy in advanced non-small cell lung cancers that developed extra-central nervous system oligoprogressive disease during EGFR tyrosine kinase inhibitor treatment: A pilot study	Mets data did not meet inclusion criteria
**Nicoś, M. 2016**	Sensitive methods for screening of the MEK1 gene mutations in patients with central nervous system metastases of non-small cell lung cancer	With incomplete data

**Nicoś, M. 2016**	PIK3CA mutations detected in patients with central nervous system metastases of non-small cell lung cancer	All brain metastases
**Nicoś, M. 2017**	Evaluation of ALK gene rearrangement in central nervous system metastases of non-small-cell lung cancer using two-step RT-PCR technique	All brain metastases
**Nieder, C. 2009**	Prognostic scores in patients with brain metastases from non-small cell lung cancer	All brain metastases
**Nishio, M. 2018**	Analysis of central nervous system efficacy in the J-ALEX study of alectinib versus crizotinib in ALK-positive non-small-cell lung cancer	With incomplete data
**Noronha, V. 2024**	A Comparative Study Evaluating the Quality of Life and Survival Outcomes in Patients Receiving Chemotherapy Versus Oral Tyrosine Kinase Inhibitor in the Third Line and Beyond Setting for Advanced NSCLC	With incomplete data
**O'Byrne, K. 2018**	Central nervous system or systemic progression analysis for EGFR mutation-positive NSCLC patients receiving afatinib in LUX-lung 3, 6, and 7	With incomplete data
**Ohe, Y. 2018**	Safety profile and effectiveness of alectinib in the real-world surveillance study of 1251 Japanese patients with ALK-positive nonsmall cell lung cancer	With incomplete data
**Ohhara, Y. 2019**	Prognostic factors for non-small cell lung cancer patients with driver mutation negative and brain metastases (HOT 1701)	With incomplete data
**Omuro, A. M. 2005**	High incidence of disease recurrence in the brain and leptomeninges in patients with nonsmall cell lung carcinoma after response to gefitinib	Selective population - Recurrent
**Ortuzar, W. 2012**	Brain metastases as the primary site of relapse in two randomized phase III pemetrexed trials in advanced non-small-cell lung cancer	Mutation-free
**Parikh, R. B. 2014**	Definitive primary therapy in patients presenting with oligometastatic non-small cell lung cancer	Mutation-free, mets data did not meet inclusion criteria
**Park, S. J. 2012**	Efficacy of epidermal growth factor receptor tyrosine kinase inhibitors for brain metastasis in non-small cell	All brain metastases

	lung cancer patients harboring either exon 19 or 21 mutation	
**Patil, S. 2019**	Management of leptomeningeal metastases in non-small cell lung cancer	Mutation-free
**Patil, T. 2019**	Targeted therapies for ROS1-rearranged non-small cell lung cancer	Mets data did not meet inclusion criteria, Only patients with progressive disease are included, and incidence cannot be calculated
**Patil, T. 2018**	The Incidence of Brain Metastases in Stage IV ROS1-Rearranged Non-Small Cell Lung Cancer and Rate of Central Nervous System Progression on Crizotinib	With incomplete data
**Petersen, I. 2000**	Chromosomal imbalances in brain metastases of solid tumors	Data does not meet inclusion criteria
**Petrovic, M. 2011**	Risk factors for brain metastases in surgically staged IIIA non-small cell lung cancer patients treated with surgery, radiotherapy and chemotherapy	Mutation-free
**Petrovic, M. 2009**	Risk factors for brain metastases after definitive chemoradiation for locally advanced non-small cell lung cancer	Mutation-free
**Piffko, A. 2022**	Clinical determinants impacting overall survival of patients with operable brain metastases from non-small cell lung cancer	All brain metastases
**Pinto, I. G. 2015**	A retrospective analysis alk-positive non-small-cell lung carcinoma (NSCLC)	With incomplete data
**Popat, S. 2018**	Intracranial efficacy of brigatinib (BRG) vs crizotinib (CRZ) in the phase III ALTA-1L trial	With incomplete data
**Pöttgen, C. 2015**	Brain relapses in stage III NSCLC after multimodality treatment: prognostic factors from a randomised trial	Mutation-free
**Press, R. H. 2017**	Kras mutant non-small cell lung cancer is associated with increased risk of salvage whole brain radiation after stereotactic radiosurgery for brain metastases	With incomplete data

**Preusser, M. 2013**	ALK gene translocations and amplifications in brain metastases of non-small cell lung cancer	Mets data did not meet inclusion criteria
**Qian, H. 2023**	Effects of concurrent TP53 mutations on the efficacy and prognosis of targeted therapy for advanced EGFR mutant lung adenocarcinoma	With incomplete data
**Ramotar, M. 2020**	Neurological Death is Common in Patients With EGFR Mutant Non-Small Cell Lung Cancer Diagnosed With Brain Metastases	All brain metastases
**Remon, J. 2024**	Perioperative Treatment Strategies in EGFR-Mutant Early-Stage NSCLC: Current Evidence and Future Challenges	With incomplete data
**Russo, A. 2015**	Epidermal Growth Factor Receptor mutational status predicts patterns of metastatic spread in treatment-naïve Adenocarcinomas of the lung	With incomplete data
**Sa, H. L. 2023**	A real-world study of the efficacy and safety of furmonertinib for patients with non-small cell lung cancer with EGFR exon 20 insertion mutations	With incomplete data
**Saad, A. G. 2008**	Immunohistochemical markers associated with brain metastases in patients with nonsmall cell lung carcinoma	Mutation-free
**Sabari, J. K. 2022**	Activity of Adagrasib (MRTX849) in Brain Metastases: Preclinical Models and Clinical Data from Patients with KRASG12C-Mutant Non-Small Cell Lung Cancer	With incomplete data
**Sandler, A. 2012**	An evidence-based review of the incidence of CNS bleeding with anti-VEGF therapy in non-small cell lung cancer patients with brain metastases	Data does not meet inclusion criteria
**Seike, T. 2011**	Interaction between lung cancer cells and astrocytes via specific inflammatory cytokines in the microenvironment of brain metastasis	With incomplete data
**Shang, K. 2022**	Efficacy and safety analyses of epidermal growth factor receptor tyrosine kinase inhibitors combined with chemotherapy in the treatment of advanced non-small-cell lung cancer with an EGFR/TP53 co-mutation	With incomplete data
**Sharma, M. 2018**	Patterns of Central Nervous System Metastases in EGFR Mutated or ALK Rearranged Non Small Cell Lung Cancer Patients	With incomplete data

**Shaw, A. T. 2017**	Lorlatinib in non-small-cell lung cancer with ALK or ROS1 rearrangement: an international, multicentre, open-label, single-arm first-in-man phase 1 trial	With incomplete data
**Shen, C. I. 2019**	Clinical outcomes of leptomeningeal metastases in EGFR-mutant lung adenocarcinoma	With incomplete data
**Shi, W. 2016**	CNS Metastases in Patients With Non-Small-Cell Lung Cancer and ALK Gene Rearrangement	All brain metastases
**Shi, Y. 2023**	OA03.05 A Randomized, Phase 3 Study of Iruplinalkib (WX-0593) vs Crizotinib in Locally Advanced or Metastatic ALK+ Non-small Cell Lung Cancer (NSCLC)	With incomplete data
**Shun, L. 2018**	Osimertinib Maintenance After Definitive Chemoradiation in Patients with Unresectable EGFRm-Positive Stage III NSCLC (LAURA)	With incomplete data
**Simionato, F. 2021**	P48.07 Real-World Impact of Upfront Osimertinib in Reducing Health Resource Utilization by Preventing Brain Metastases	With incomplete data
**Sivignon, M. 2020**	Cost-effectiveness of alectinib compared to crizotinib for the treatment of first-line ALK+ advanced non-small-cell lung cancer in France	With incomplete data
**Soo, R. A. 2024**	TARGET: A Phase II, Open-Label, Single-Arm Study of 5-Year Adjuvant Osimertinib in Completely Resected EGFR-Mutated Stage II to IIIB NSCLC Post Complete Surgical Resection	With incomplete data
**Soon, Y. Y. 2023**	OA22.06 Changes in Cognition after Osimertinib, with or without SRS, in EGFRm NSCLC with Brain Metastases: a Pooled Analysis of RCTs	With incomplete data
**Sperduto, P. W. 2016**	The Effect of Gene Alterations and Tyrosine Kinase Inhibition on Survival and Cause of Death in Patients With Adenocarcinoma of the Lung and Brain Metastases	With incomplete data
**Spina, R. 2013**	Outcomes of chemoradiation for patients with locally advanced non-small-cell lung cancer	Mutation-free
**Sreter, K. 2017**	Brain metastasis and epidermal growth factor receptor mutations in Croatian caucasians with lung adenocarcinoma	With incomplete data

**Sreter, K. B. 2017**	EGFR mutations in lung adenocarcinoma and brain metastases: A Croatian single institution experience	With incomplete data
**Su, P. L. 2021**	First-line treatment with irreversible tyrosine kinase inhibitors associated with longer OS in EGFR mutation-positive non-small cell lung cancer	With incomplete data
**Sui, J. S. 2023**	Brain Metastases in Patients with Fusion-Positive Lung Cancers	With incomplete data
**Sun, Y. 2020**	Evaluation of radiotherapy combined with targeted therapy and concurrent radiotherapy, chemotherapy in the treatment of Non-Small Cell Lung Cancer with brain metastasis	Mutation-free
**Sung, S. 2018**	Intracranial control and survival outcome of tyrosine kinase inhibitor (TKI) alone versus TKI plus radiotherapy for brain metastasis of epidermal growth factor receptor-mutant non-small cell lung cancer	All brain metastases
**Takeyasu, Y. 2022**	Lorlatinib Versus Pemetrexed-Based Chemotherapy in Patients With ALK-rearranged NSCLC Previously Treated With Alectinib	Mets data did not meet inclusion criteria
**Tang, W. H. 2015**	Prognostic factors in patients with non-small cell lung carcinoma and brain metastases: a Malaysian perspective	All brain metastases
**Tatineni, V. 2023**	First- versus Third-Generation EGFR Tyrosine Kinase Inhibitors in EGFR-Mutated Non-Small Cell Lung Cancer Patients with Brain Metastases	All brain metastases
**Tatineni, V. 2023**	Combination of EGFR-Directed Tyrosine Kinase Inhibitors (EGFR-TKI) with Radiotherapy in Brain Metastases from Non-Small Cell Lung Cancer: A 2010-2019 Retrospective Cohort Study	All brain metastases
**Teich, V. 2019**	PCN221 a multicriteria decision analysis (mcda) to evaluate alternative treatments for locally advanced or metastatic EGFR positive non-small cell lung cancer under the brazilian private healthcare system perspective	With incomplete data
**Thomas, R. 2023**	Molecular epidemiology and clinical characteristics of epidermal growth factor receptor mutations in NSCLC: A single-center experience from India	With incomplete data

**Tibdewal, A. 2023**	Upfront or Delayed Cranial RT in Oncogene Mutated NSCLC with Asymptomatic Brain Metastases: A Phase III Randomized Controlled Trial	With incomplete data
**Tozuka, T. 2019**	P2.14-41 Risk Factors for Brain Metastasis in Patients with EGFR Mutant Non-Small Cell Lung Cancer	With incomplete data
**Varlotto, J. M. 2022**	Bevacizumab's Association With a Decreased Risk of Brain Metastases in ECOG-ACRIN E1505, a Phase 3 Randomized Trial of Adjuvant Chemotherapy With or Without Bevacizumab in Surgically Resected NSCLC	Mutation-free
**Villalva, C. 2013**	EGFR, KRAS, BRAF, and HER-2 molecular status in brain metastases from 77 NSCLC patients	All brain metastases
**Wadhwa, S. 2019**	Radiogenomic signatures of NSCLC brain metastases: A potential non-invasive imaging marker for ALK mutation	With incomplete data
**Wakuda, K. 2020**	A phase II study of Osimertinib for patients with radiotherapy-naive CNS metastasis of non-small cell lung cancer: treatment rationale and protocol design of the OCEAN study (LOGIK 1603/WJOG 9116L)	All brain metastases
**Wang, G. 2019**	Distribution of brain metastasis from lung cancer	All brain metastases
**Wang, J. 2019**	EP1.14-49 A Phase IIIb Open-Label Study of Afatinib in EGFR TKI-Naïve Patients with EGFRm+ NSCLC: Exploratory Biomarker Analysis	With incomplete data
**Wang, Q. 2022**	Improved Survival With Surgical Treatment of Primary Lung Lesions in Non-Small Cell Lung Cancer With Brain Metastases: A Propensity-Matched Analysis of Surveillance, Epidemiology, and End Results Database	All brain metastases
**Wang, R. 2023**	An effective prognostic model for assessing prognosis of non-small cell lung cancer with brain metastases	With incomplete data
**Wang, T. J. 2015**	Does lung cancer mutation status and targeted therapy predict for outcomes and local control in the setting of brain metastases treated with radiation?	All brain metastases
**Wu, W. S. 2013**	The epidermal growth factor receptor-tyrosine kinase inhibitor era has changed the causes of death of patients with advanced non-small-cell lung cancer	Mets data did not meet inclusion criteria

**Wu, Y. 2018**	A Phase IIIb Trial of Afatinib in EGFRm+ NSCLC: Analyses of Outcomes in Patients with Brain Metastases or Dose Reductions	With incomplete data
**Wu, Y. L. 2022**	Capmatinib plus osimertinib versus platinum-pemetrexed doublet chemotherapy as second-line therapy in patients with stage IIIb/IIIc or IV EGFR-mutant, T790 Mnegative NSCLC harboring MET amplification	With incomplete data
**Xie, L. 2019**	Osimertinib for EGFR-Mutant Lung Cancer with Brain Metastases: Results from a Single-Center Retrospective Study	All brain metastases
**Xu, T. 2023**	Development and validation of a nomogram for predicting the overall survival in non-small cell lung cancer patients with liver metastasis	All brain metastases
**Xu, X. 2024**	Risk factors for brain metastases in locally advanced non-small cell lung cancer patients treated with radical radiotherapy	Mutation-free
**Xu, Y. 2016**	Epidermal growth factor tyrosine kinase inhibitors used in the treatment of NSCLC patients with leptomeningeal metastasis	All brain metastases
**Yamaguchi, H. 2022**	Osimertinib for RT-naïve CNS metastasis of EGFR mutation-positive NSCLC: Phase II OCEAN study, part of the first-line cohort	With incomplete data
**Yamaguchi, M. 2017**	Pulmonary Resection for Synchronous M1b-cStage IV Non-Small Cell Lung Cancer Patients	Mutation-free
**Yan, W. 2019**	Whole brain radiation therapy does not improve the overall survival of EGFR-mutant NSCLC patients with leptomeningeal metastasis	All brain metastases
**Yang, B. 2019**	Incidence of brain metastasis in lung adenocarcinoma at initial diagnosis on the basis of stage and genetic alterations	With incomplete data
**Yang, H. 2023**	P1.11-03 Proteomics-based Predictive Model for the Increased Brain Metastasis Risk in Resected Lung Adenocarcinoma with EGFR Mutaion	With incomplete data
**Kwon, J. 2014**	Impact of multimodality approach for patients with leptomeningeal metastases from solid tumors	No genotypic changes and with incomplete data

**Lee, B. 2023**	Efficacy of lazertinib for symptomatic or asymptomatic brain metastases in treatment-naive patients with advanced EGFR mutation-positive non-small cell lung cancer: Protocol of an open-label, single-arm phase II trial	With incomplete data
**Lee, J. 2019**	High Incidence of CNS Metastases in Advanced or Recurrent Non-Small Cell Lung Cancer Patients with RET Fusion	With incomplete data
**Lee, J. 2020**	Characteristics and outcomes of RET-rearranged Korean non-small cell lung cancer patients in real-world practice	With incomplete data
**Lee, J. S. 2019**	The impact of systemic treatment on brain metastasis in patients with non-small-cell lung cancer: A retrospective nationwide population-based cohort study	With incomplete data
**Lee, Y. 2013**	Impact of EGFR tyrosine kinase inhibitors versus chemotherapy on the development of leptomeningeal metastasis in never smokers with advanced adenocarcinoma of the lung	With incomplete data
**Leyrat, B. 2022**	Local control and radionecrosis of brain metastases from non-small-cell lung cancer treated by hypofractionated stereotactic radiotherapy: Evaluation of predictive factors	With incomplete data
**Li, A. 2020**	Prognostic value of lymphocyte-to-monocyte ratio and systemic immune-inflammation index in non-small-cell lung cancer patients with brain metastases	The research content is not related to the effect of gene mutation on brain metastasis.
**Li, J. 2021**	Downregulation of lncRNA XR_429159.1 Linked to Brain Metastasis in Patients With Limited-Stage Small-Cell Lung Cancer	With incomplete data
**Li, N. 2023**	Real-world treatment and prognostic factors for survival in ALK plus non-small cell lung cancer (NSCLC) patients with brain metastases in China	With incomplete data
**Li, N. 2015**	Randomized phase III trial of prophylactic cranial irradiation versus observation in patients with fully resected stage IIIA-N2 nonsmall-cell lung cancer and high risk of cerebral metastases after adjuvant chemotherapy	With incomplete data

**Li, Q. X. 2013**	Associations between Single-Nucleotide Polymorphisms in the PI3K-PTEN-AKT-mTOR Pathway and Increased Risk of Brain Metastasis in Patients with Non-Small Cell Lung Cancer	With incomplete data
**Lin, C. C. 2020**	Targeting positive feedback between BASP1 and EGFR as a therapeutic strategy for lung cancer progression	The research content is not related to the effect of gene mutation on brain metastasis.
**Lin, C. H. 2015**	Increased survival with the combination of stereotactic radiosurgery and gefitinib for non-small cell lung cancer brain metastasis patients: a nationwide study in Taiwan	The research content is not related to the effect of gene mutation on brain metastasis.
**Lin, J. H. 2017**	The association between clinical prognostic factors and epidermal growth factor receptor-tyrosine kinase inhibitor (EGFR-TKI) efficacy in advanced non-small-cell lung cancer patients: a retrospective assessment of 94 cases with EGFR mutations	With incomplete data
**Liu, H. L. 2020**	Clinical study on different doses and fractionated radiotherapies for multiple brain metastases of non-EGFR mutant lung adenocarcinoma	The research content is not related to the effect of gene mutation on brain metastasis.
**Liu, K. 2020**	Icotinib is as efficacious as gefitinib for brain metastasis of EGFR mutated non-small-cell lung cancer	The research content is not related to the effect of gene mutation on brain metastasis.
**Liu, Y. 2021**	The Efficacy of First-Generation EGFR-TKI Combined With Brain Radiotherapy as the First-Line Treatment for Lung Adenocarcinoma Patients With Brain Metastases and EGFR Sensitive Mutations: A Retrospective study	The data collected were for lung adenocarcinoma
**London, D. 2022**	The incidence and predictors of new brain metastases in patients with non-small cell lung cancer following discontinuation of systemic therapy	With incomplete data
**Luo, D. 2014**	EGFR mutation status and its impact on survival of Chinese non-small cell lung cancer patients with brain metastases	Selective population
**Yang, J. C. H. 2018**	Competing central nervous system or systemic progression analysis for patients with EGFR mutation-	With incomplete data

	positive NSCLC receiving afatinib in LUX-Lung 3, 6, and 7	
**Yano, S. 2000**	Expression of vascular endothelial growth factor is necessary but not sufficient for production and growth of brain metastasis	With incomplete data
**Yomo, S. 2018**	Impacts of EGFR-mutation status and EGFR-TKI on the efficacy of stereotactic radiosurgery for brain metastases from non-small cell lung adenocarcinoma: A retrospective analysis of 133 consecutive patients	With incomplete data
**Yomo, S. 2019**	The impact of EGFR-TKI use on clinical outcomes of lung adenocarcinoma patients with brain metastases after Gamma Knife radiosurgery: a propensity score-matched analysis based on extended JLGK0901 dataset	With incomplete data
**Yu, H. 2019**	Chinese perspectives on clinical efficacy and safety of alectinib in patients with ALK-positive advanced non-small cell lung cancer	With incomplete data
**Yu, H. A. 2017**	Phase 1 study of twice weekly pulse dose and daily low-dose erlotinib as initial treatment for patients with EGFR-mutant lung cancers	With incomplete data
**Yuan, R 2016**	Radiographic patterns and survival of patients with early and late brain metastases in EGFR wild type and mutant non small cell lung cancer	With incomplete data
**Yun, P. J. 2019**	Brain metastases in resected non-small cell lung cancer: The impact of different tyrosine kinase inhibitors	With incomplete data
**Zhang, Q. 2015**	Risk factors of brain metastases in completely resected stage IIIa(N2) non-small cell lung cancer	With incomplete data
**Zhang, Q. 2020**	Comparison of the characteristics of uncommon epidermal growth factor receptor (EGFR) mutations and EGFR-tyrosine kinase inhibitor treatment in patients with non-small cell lung cancer from different ethnic groups	With incomplete data
**Zhao, X. 2014**	Efficacy of icotinib versus traditional chemotherapy as first-line treatment for preventing brain metastasis from advanced lung adenocarcinoma in patients with epidermal growth factor receptor-sensitive mutation	With incomplete data

**Zhao, Y. 2019**	Management of Central Nervous System Metastases in Patients With Advanced Anaplastic Lymphoma Kinase-Rearranged Non-Small-Cell Lung Cancer During Crizotinib Treatment	With incomplete data
**Zhuang, H. 2020**	Tyrosine Kinase Inhibitor Resistance Increased the Risk of Cerebral Radiation Necrosis After Stereotactic Radiosurgery in Brain Metastases of Non-small-Cell Lung Cancer: A Multi-Institutional Retrospective Case-Control Study	With incomplete data
**Mitra, D. 2019**	EGFR mutant locally advanced non-small cell lung cancer is at increased risk of brain metastasis	With incomplete data
**Vanita,N.2024**	Clinical characteristics, outcomes and prognostic factors in KRAS mutant lung cancers: experience from a tertiary care cancer center in India	With incomplete data
**Novello, S. 2018**	Alectinib versus chemotherapy in crizotinib-pretreated anaplastic lymphoma kinase positive non-small-cell lung cancer: results from the phase III ALUR study	With incomplete data
**Patil, T. 2018**	The Incidence of Brain Metastases in Stage IV ROS1-Rearranged Non-Small Cell Lung Cancer and Rate of Central Nervous System Progression on Crizotinib	With incomplete data
**Aiko, N. 2018**	Comparison of the efficacies of first-generation epidermal growth factor receptor tyrosine kinase inhibitors for brain metastasis in patients with advanced non-small-cell lung cancer harboring EGFR mutations	With incomplete data
**Beypinar, I. 2019**	The relationship between EGFR mutation and metastasis pattern in lung adenocarcinoma	With incomplete data
**Bhatt, V. R. 2017**	Epidermal growth factor receptor mutational status and brain metastases in non–small-cell lung cancer	With incomplete data
**Dormieux, A. 2020**	Association of metastatic pattern and molecular status in stage IV non-small cell lung cancer adenocarcinoma	With incomplete data
**Fujimoto, D. 2014**	Features and prognostic impact of distant metastasis in patients with stage IV lung adenocarcinoma harboring EGFR mutations: importance of bone metastasis	With incomplete data
**Guan, J. 2016**	EGFR mutations are associated with higher incidence of distant metastases and smaller tumor size in patients with non-small-cell lung cancer based on PET/CT scan	With incomplete data

**Hsu, F. 2017**	Patterns of spread and prognostic implications of lung cancer metastasis in an era of driver mutations	With incomplete data
**Iuchi, T. 2015**	Frequency of brain metastases in non-small-cell lung cancer, and their association with epidermal growth factor receptor mutations	With incomplete data
**Jiang, X. 2016**	[Clinical Analysis of Icotinib on Beneficiary of Advanced Non-small Cell Lung Cancer with EGFR Common Mutation]	With incomplete data
**Jung, H. A. 2020**	The different central nervous system efficacy among gefitinib, erlotinib and afatinib in patients with epidermal growth factor receptor mutation-positive non-small cell lung cancer	With incomplete data
**Kashima, J. 2016**	Survival of patients with brain metastases from non-small cell lung cancer harboring EGFR mutations treated with epidermal growth factor receptor tyrosine kinase inhibitors	With incomplete data
**Kwon, B. S. 2020**	Impact of clinicopathologic features on leptomeningeal metastasis from lung adenocarcinoma and treatment efficacy with epidermal growth factor receptor tyrosine kinase inhibitor	With incomplete data
**Lau, S. C. 2019**	Outcome Differences Between First- and Second-generation EGFR Inhibitors in Advanced EGFR Mutated NSCLC in a Large Population-based Cohort	With incomplete data
**Li, B. 2015**	The correlation between EGFR mutation status and the risk of brain metastasis in patients with lung adenocarcinoma	With incomplete data
**Li, Y. S. 2020**	Association of Cerebrospinal Fluid Tumor DNA Genotyping with Survival among Patients with Lung Adenocarcinoma and Central Nervous System Metastases	With incomplete data
**Yu, H. A. 2020**	Effect of Osimertinib and Bevacizumab on Progression-Free Survival for Patients With Metastatic EGFR-Mutant Lung Cancers: A Phase 1/2 Single-Group Open-Label Trial	With incomplete data
**Yu, H. A. 2013**	Local therapy with continued EGFR tyrosine kinase inhibitor therapy as a treatment strategy in EGFR-mutant advanced lung cancers that have developed acquired resistance to EGFR tyrosine kinase inhibitors	With incomplete data

**Zhang, Y. 2020**	Detection of Nonreciprocal/Reciprocal ALK Translocation as Poor Predictive Marker in Patients With First-Line Crizotinib-Treated ALK-Rearranged NSCLC	With incomplete data
**Zhang, Y. 2020**	Detection of Nonreciprocal/Reciprocal ALK Translocation as Poor Predictive Marker in Patients With First-Line Crizotinib-Treated ALK-Rearranged NSCLC	With incomplete data
**Dormieux, A. 2021**	Association of metastatic pattern and molecular status in stage IV non-small cell lung cancer adenocarcinoma	With incomplete data
**Johung, K. L. 2016**	Extended Survival and Prognostic Factors for Patients With ALK-Rearranged Non-Small-Cell Lung Cancer and Brain Metastasis	With incomplete data
**Rangachari, D. 2016**	Brain metastases in patients with EGFR-mutated or ALK-rearranged non-small-cell lung cancers	With incomplete data
**Wang, H. 2019**	EP1.14-09 Driver Genes as Predictive Indicators of Brain Metastasis in Patients with Advanced NSCLC: EGFR and ALK as Well as RET Gene Mutations	With incomplete data
**Dormieux, A. 2020**	Association of metastatic pattern and molecular status in stage IV non-small cell lung cancer adenocarcinoma	With incomplete data
**Wang, H. 2019**	EP1.14-07 Driver Genes as Predictive Indicators of Brain Metastasis in Patients with Advanced NSCLC: EGFR and ALK as Well as RET Gene Mutations	With incomplete data
**Dormieux, A. 2023**	Association of metastatic pattern and molecular status in stage IV non-small cell lung cancer adenocarcinoma	With incomplete data
**Lamberti, G. 2023**	Incidence of Brain Metastases and Preliminary Evidence of Intracranial Activity With Sotorasib in Patients With KRASG12C-Mutant Non-Small-Cell Lung Cancer	With incomplete data
**Kris, M. G. 2018**	Frequency of brain metastases and outcomes in patients with HER2-, KRAS-, and EGFRmutant lung cancers	With incomplete data

Supplementary Table 6. Baseline demographics and prevalence rates for studies included.

Author and year	Stages included	Sample Size	Number with baseline mets	Baseline prevalence (%)
**EGFR**				
Ahn, M. J. 2020	stage III-IV	66	20	30.30
Aiko, N. 2018	stage III-IV	77	27	35.06
Akamatsu, H. 2014	stage III-IV	13	0	0
Auliac, J. B. 2019	stage III-IV	205	87	42.44
Baek, M. Y. 2018	stage III-IV	73	7	9.59
Byeon, S. 2018	stage III-IV	27	11	40.74
Beypinar, I. 2019	unstaged information	27	5	18.52
Bi, J. 2016	mixed	108	28	25.93
Chen, L. M. 2019	unstaged information	74	53	71.62
Chooback, N. 2018	stage III-IV	499	89	17.84
Cui, Q. L. 2022	stage III-IV	96	34	35.42
Dormieux, A. 2020	stage III-IV	135	48	35.56
Dube-Pelletier. 2018	stage III-IV	11	1	9.09
Fujimoto, D. 2014	stage III-IV	98	45	45.92
Ge. 2017	stage III-IV	100	28	28.00
Guan, J. 2016	stage III-IV	85	25	29.41
Gutiérrez, L. 2021	stage III-IV	505	85	16.83
Han, G. 2016	mixed	103	39	37.86
Hendriks, L. 2013	stage III-IV	74	23	31.08
Hendriks, L. E. 2014	unstaged information	68	27	39.71
Heon, S. 2010	stage III-IV	100	19	19.00
Ho, G. F. 2019	stage III-IV	85	25	29.41
Hsu, F. 2017	stage III-IV	121	29	23.97

Jiang, X. 2016	stage III-IV	231	59	25.54
Joo, J. W. 2018	mixed	331	18	5.44
Jung, H. A. 2020	unstaged information	559	198	35.42
Jung, H. A. 2023	stage III-IV	30	3	10.00
Kashima, J. 2016	stage III-IV	200	62	31.00
Kim, J. 2023	stage III-IV	703	262	37.27
Kris, M. G. 2018	unstaged information	111	48	43.24
Kwon, B. S. 2020	unstaged information	1189	410	34.48
Lampaki, S. 2022	mixed	180	34	18.89
Lau, S. C. 2019	unstaged information	484	99	20.45
Lin, C. 2018	stage III-IV	306	87	28.43
Luchi, T. 2013	unstaged information	41	31	75.61
Luchi, T. 2015	mixed	159	25	15.72
Muttiah, C. 2017	mixed	23	5	21.74
Ng, D. Z. 2016	mixed	200	63	32.50
Nieva, J. 2022	stage III-IV	1029	325	31.58
Offin, M. 2019	stage III-IV	200	62	31.00
Ouyang. 2020	stage III-IV	226	69	30.53
Patel, S. H. 2017	mixed	189	78	41.27
Patil. 2018	stage III-IV	192	53	27.60
Popat, S. 2021	stage III-IV	191	26	13.61
Rangachari, D. 2015	stage III-IV	109	24	22.02
Sadoyama, S. 2019	mixed	16	2	12.50
Shah, R. 2021	stage III-IV	431	41	9.51
Stanic, K. 2016	mixed	137	26	18.98
Tomasini, P. 2017	stage III-IV	142	52	36.62
Tozuka, T. 2023	stage III-IV	62	17	27.42

Veerman, G. D. M. 2023	unstaged information	572	201	35.14
Wang. 2019	stage III-IV	60	24	40.00
Wang, B. 2017	stage III-IV	465	121	26.02
Wang, H. 2020	stage III-IV	226	55	24.34
Wu, Y. L. 2023	stage III-IV	77	27	35.06
Yang. 2019	stage III-IV	98	39	39.80
Yang, B. 2019	stage III-IV	96	52	54.17
Yang, H. 2020	stage III-IV	516	207	40.12
Yoshida. 2019	stage III-IV	170	43	25.29
Yu, M. 2018	mixed	99	42	42.42
**ALK**				
Burudpakdee. 2018	stage III-IV	181	49	27.07
Chu, L. 2019	unstaged information	40	27	67.50
Deng, H. 2019	stage III-IV	47	12	25.53
Dormieux, A. 2020	stage III-IV	81	34	41.98
Dube-Pelletier. 2021	unstaged information	3	2	66.67
Gadgeel. 2018	stage III-IV	89	7	7.87
Gainor. 2017	mixed	196	77	39.29
Johung, K. L. 2016	mixed	90	27	30.00
Nakamura, T. 2023	stage I-II	42	12	28.57
Ni, J. 2019	mixed	93	35	37.63
Nishio, M. 2017	mixed	46	14	30.43
Ou, S. H. I. 2016	stage III-IV	138	84	60.87
Rangachari, D. 2015	stage III-IV	21	5	23.81
Patil MD. 2018	mixed	115	39	33.91
Shaw, A. T. 2020	stage III-IV	149	38	25.50

Shi, Y. 2024	stage III-IV	149	44	29.53
Solomon, B. J. 2022	stage III-IV	296	78	26.35
Soria, J. C. 2017	stage III-IV	376	121	32.18
Wang, H. 2020	stage III-IV	22	4	18.18
Yang. 2019	unstaged information	16	3	18.75
Yang, H. 2020	stage III-IV	76	24	31.58
Zhang, Y. 2020	stage III-IV	112	21	18.75
**BRAF**				
Dagogo-Jack, I. 2019	mixed	236	139	58.90
Dormieux, A. 2020	stage III-IV	47	10	21.28
Wang, H. 2019	stage III-IV	4	0	0
**HER2**				
Ahn, B. C. 2023	mixed	44	14	31.82
Offin. 2019	stage III-IV	98	19	19.39
**KRAS**				
Calles. 2015	unstaged information	126	41	32.54
Dormieux, A. 2020	stage III-IV	141	51	36.17
Hendriks, L. E. 2014	unstaged information	65	9	13.85
Kris, M. G. 2018	stage III-IV	200	48	24.00
Noronha, V. 2024	mixed	133	17	12.78
Offin, M. 2019	stage III-IV	200	48	24.00
Patil. 2018	unstaged information	102	29	28.43
Swart, E. M. 2023	stage III-IV	153	30	19.61
Tomasini, P. 2016	stage III-IV	47	18	38.30
Wang, H. 2020	stage III-IV	55	6	10.91
Yang. 2019	unstaged information	13	6	46.15

**MET**				
Offin, M. 2020	stage III-IV	83	14	16.87
Wang, H. 2019	stage III-IV	3	0	0
**RET**				
Drilon. 2018	stage III-IV	185	46	24.86
Lee. 2020	mixed	59	17	28.81
Murciano. 2023	stage III-IV	61	30	49.18
Wang, H. 2020	mixed	11	7	63.64
**ROS1**				
Ai, X. 2021	stage III-IV	37	0	0
Gainor. 2017	mixed	39	10	25.64
Nakamura, T. 2023	stage I-II	26	3	11.54
Park, S. 2018	mixed	103	23	22.33
Patil. 2018	unstaged information	33	12	36.36
Wang, H. 2020	mixed	3	0	0

Supplementary Table 7. Baseline demographics and incidence rates for studies included.

Author and year	Stages included	Number of patients	Number developing BM	Incidence (%)
**EGFR**				
Alzate, J. D. 2023	Unstaged information	170	38	22.35
Auliac, J. B. 2019	stageIII-IV	118	4	3.39
Baek, M. Y. 2018	stageIII-IV	66	20	30.30
Chen, L. M. 2019	mixed	21	11	52.38
Chooback, N. 2017	stageIII-IV	409	139	33.99
Cui, Q. L. 2022	stageIII-IV	62	19	30.65
Ge. 2017	stageIII	21	10	47.62
Han, G. 2016	mixed	87	17	19.54
Hendriks, L. E. 2014	unstaged information	57	15	26.32
Heon, S. 2010	stageIII-IV	81	28	34.57
Ho, G. F. 2019	stageIII-IV	60	7	11.67
Hsu, F. 2017	stageIII-IV	92	18	19.57
Jung, H. A. 2020	unstaged information	361	69	19.11
Jung, H. A. 2023	stageIII-IV	27	9	33.33
Kim, H. 2023	stageIII-IV	71	27	38.03
Kogure, Y. 2015	stageIII-IV	30	5	16.67
Kwon, B. S. 2020	stageIII-IV	117	88	75.21
Lampaki, S. 2022	mixed	146	17	11.64
Lee, H. L. 2012	mixed	43	30	69.77
Lee, K. 2021	mixed	64	23	35.94
Li, C. 2020	mixed	332	135	40.66

Li, C. 2021	stageIII-IV	135	54	40.00
Li, C. H. 2019	stageIII-IV	100	30	30.00
Liang, S. K. 2018	mixed	259	82	31.66
Lin, C. 2018	stageIII-IV	219	111	50.68
Lin, J. H. 2021	stageIII-IV	21	6	28.57
Liu, J. 2016	stageIII-IV	258	161	62.40
Ma, X. 2016	mixed	134	34	25.37
Muttiah, C. 2017	mixed	18	1	5.56
Ng, D. Z. 2016	mixed	137	40	29.20
Nieva, J. 2022	stageIII-IV	704	155	22.02
Nishie, K. 2012	mixed	64	16	25.00
Offin. 2022	stageIII-IV	138	32	23.19
Ouyang, W. 2020	stageIII-IV	157	30	19.11
Ouyang, W. 2020	stageIII-IV	157	30	19.11
Patel, S. H. 2017	mixed	111	34	30.63
Popat, S. 2021	stageIII-IV	165	29	17.58
Rangachari, D. 2015	stageIII-IV	75	29	38.67
Sadoyama, S. 2019	mixed	14	9	64.29
Shah, R. 2021	stageIII-IV	26	11	42.31
Stanic, K. 2016	mixed	111	21	18.92
Tomasini, P. 2017	stageIII-IV	8	6	75.00
Tozuka, T. 2023	stageIII-IV	45	7	15.56
Veerman, G. D. M. 2023	Unstaged information	371	254	68.46
Wang, B. X. 2017	stageIII-IV	1254	148	11.80
Wang, H. 2020	stageIII-IV	171	22	12.87

Wu, Y. L. 2023	stageIII-IV	50	16	32.00
Yang, H. 2021	stageIII-IV	309	160	51.78
Yao, Z. H. 2017	stageIII-IV	226	63	27.88
Yoshida. 2019	stageIII-IV	127	27	21.26
Yu, M. 2018	mixed	48	16	33.33
Yu, X. 2021	mixed	571	89	15.59
Zhai, X. Y. 2021	stageIII-IV	61	33	54.10
Zhao, Y. 2022	mixed	367	148	40.33
Zhao. 2016	unstaged information	396	99	25.00
Zhou, F. 2023	stageIII-IV	366	129	35.25
Zhou, S. 2024	stageIII-IV	38	34	89.47
Zhu, H. 2017	mixed	134	34	25.37
**ALK**				
Burudpakdee. 2018	stageIII-IV	132	35	26.52
Deng, H. 2019	stage III-IV	35	9	25.71
gadgeel. 2018	stageIII-IV	81	6	7.41
Gainor.2017	mixed	196	110	56.12
Lara-Mejía, L. 2024	stage III-IV	116	35	30.17
Lee, K. 2021	mixed	6	1	16.67
Ni, J. 2019	mixed	58	13	22.41
Ou, S. H. I. 2016	stage III-IV	54	33	61.11
Patil MD.2018	mixed	115	39	33.91
Rangachari. 2015	stage III-IV	16	9	56.25
Shaw, A. T. 2020	stage III-IV	111	41	36.94
Shi, Y. 2024	stage III-IV	106	10	9.43

Shi, Y. 2024	stage III-IV	105	19	18.10
Sivignon. 2020	stageIII-IV	228	57	25.00
Solomon, B. J. 2022	stage III-IV	218	20	9.17
Soria, J. C. 2017	stage III-IV	81	25	30.86
Wang, H. 2021	stage III-IV	18	5	27.78
Yang, H. 2022	stage III-IV	52	20	38.46
**BRAF**				
Wang, H. 2019	stage III-IV	4	2	50.00
Dagogo-Jack, I. 2019	mixed	209	11	5.26
Patil MD.2018	mixed	16	3	18.75
**KRAS**				
Swart, E. M. 2023	stage III-IV	123	56	45.53
Tomasini, P. 2016	stage III-IV	29	8	27.59
Wang, H. 2019	mixed	49	6	12.24
Hendriks, L. E. 2014	unstaged information	56	14	25.00
Offin, M. 2019	stage III-IV	152	16	10.53
**HER2**				
Yang, S. 2021	stage III-IV	98	14	14.29
Ahn, B. C. 2023	mixed	30	10	33.33
**ROS1**				
Ai, X. 2021	stage III-IV	33	4	12.12
Park, S. 2018	mixed	80	24	30.00
Patil MD.2018	mixed	8	6	75.00
Li, Z. 2018	mixed	36	6	16.67
Wang, H. 2019	mixed	3	0	0

Gainor.2017	mixed	29	7	24.14
**MET**				
Offin, M. 2020	stage III-IV	69	16	23.19
Wang, H. 2019	stage III-IV	3	1	33.33
**RET**				
Drilon.2018	stageIII-IV	139	64	46.04
Lee.2020	mixed	42	11	26.19
Murciano. 2023	stageIII-IV	31	0	0
Wang, H. 2019	mixed	4	0	0

Supplementary Table 8. Cumulative incidence and prevalence for less commonly reported genomic mutations (PI3K, HER-2, MET-14, FGFR1, and BRAF).

	Included studies (Number of patients)	Prevalence (%)	Median follow-up (months)	Incidence per patient-year
**Incidence (total)**				
**ERCC1**	1(663)	98(15)	56.0	0.03
**ERBB2**	1(7)	1(14)	41.0	0.04
**BRAF**	1(4)	2(50)	41.0	0.15
**Prevalence**				
	**Included studies (Number of patients)**	**Prevalence (%)**		
**BRAF**	1(4)	2(50)		
**PI3K**	1 (33)	9 (27.3)		
**FGFR1**	1 (16)	0 (0.0)		
**ERBB2**	1(7)	0 (0.0)		

Supplementary Figures

Supplementary Figure 1. Forest plot of brain metastases prevalence in EGFR+ NSCLC (N=60).

Study	Events	Total	Proportion [95% CI]	Weight
Ahn, M. J. 2020	20	66	0.303 [0.196; 0.429]	1.6%
Aiko, N. 2018	27	77	0.351 [0.245; 0.468]	1.6%
Akamatsu, H. 2014	0	13	0.000 [0.000; 0.247]	1.6%
Auliac, J. B. 2019	87	205	0.424 [0.356; 0.495]	1.7%
Baek, M. Y. 2018	7	73	0.096 [0.039; 0.188]	1.7%
Byeon, S. 2018	11	27	0.407 [0.224; 0.612]	1.2%
Beypinar, I. 2019	5	27	0.185 [0.063; 0.381]	1.4%
Bi, J. 2016	28	108	0.259 [0.180; 0.352]	1.7%
Chen, L. M. 2019	53	74	0.716 [0.599; 0.815]	1.6%
Chooback, N. 2018	89	499	0.178 [0.146; 0.215]	1.8%
Cui, Q. L. 2022	34	96	0.354 [0.259; 0.458]	1.6%
Dormieux, A. 2020	48	135	0.356 [0.275; 0.442]	1.7%
Dube−Pelletier. 2018	1	11	0.091 [0.002; 0.413]	1.3%
Fujimoto, D. 2014	45	98	0.459 [0.358; 0.563]	1.6%
Ge. 2017	28	100	0.280 [0.195; 0.379]	1.7%
Guan, J. 2016	25	85	0.294 [0.200; 0.403]	1.6%
Gutiérrez, L. 2021	85	505	0.168 [0.137; 0.204]	1.8%
Han, G. 2016	39	103	0.379 [0.285; 0.480]	1.6%
Hendriks, L. 2013	23	74	0.311 [0.208; 0.429]	1.6%
Hendriks, L. E. 2014	27	68	0.397 [0.280; 0.523]	1.5%
Heon, S. 2010	19	100	0.190 [0.118; 0.281]	1.7%
Ho, G. F. 2019	25	85	0.294 [0.200; 0.403]	1.6%
Hsu, F. 2017	29	121	0.240 [0.167; 0.326]	1.7%
Jiang, X. 2016	59	231	0.255 [0.200; 0.317]	1.8%
Joo, J. W. 2018	18	331	0.054 [0.033; 0.085]	1.8%
Jung, H. A. 2020	198	559	0.354 [0.315; 0.395]	1.8%
Jung, H. A. 2023	3	30	0.100 [0.021; 0.265]	1.6%
Kashima, J. 2016	62	200	0.310 [0.247; 0.379]	1.7%
Kim, J. 2023	262	703	0.373 [0.337; 0.410]	1.8%
Kris, M. G. 2018	48	111	0.432 [0.339; 0.530]	1.6%
Kwon, B. S. 2020	410	1189	0.345 [0.318; 0.373]	1.8%
Lampaki, S. 2022	34	180	0.189 [0.135; 0.254]	1.8%
Lau, S. C. 2019	99	484	0.205 [0.169; 0.243]	1.8%
Lin, C. 2018	87	306	0.284 [0.234; 0.338]	1.8%
Luchi, T. 2013	31	41	0.756 [0.597; 0.876]	1.5%
Luchi, T. 2015	25	159	0.157 [0.104; 0.223]	1.8%
Muttiah, C. 2017	5	23	0.217 [0.075; 0.437]	1.3%
Ng, D. Z. 2016	63	200	0.315 [0.251; 0.384]	1.7%
Nieva, J. 2022	325	1029	0.316 [0.288; 0.345]	1.8%
Offin, M. 2019	62	200	0.310 [0.247; 0.379]	1.7%
Ouyang. 2020	69	226	0.305 [0.246; 0.370]	1.8%
Patel, S. H. 2017	78	189	0.413 [0.342; 0.486]	1.7%
Patil. 2018	53	192	0.276 [0.214; 0.345]	1.8%
Popat, S. 2021	26	191	0.136 [0.091; 0.193]	1.8%
Rangachari, D. 2015	24	109	0.220 [0.146; 0.310]	1.7%
Sadoyama, S. 2019	2	16	0.125 [0.016; 0.383]	1.3%
Shah, R. 2021	41	431	0.095 [0.069; 0.127]	1.8%
Stanic, K. 2016	26	137	0.190 [0.128; 0.266]	1.7%
Tomasini, P. 2017	52	142	0.366 [0.287; 0.451]	1.7%
Tozuka, T. 2023	17	62	0.274 [0.169; 0.402]	1.6%
Veerman, G. D. M. 2023	201	572	0.351 [0.312; 0.392]	1.8%
Wang. 2019	24	60	0.400 [0.276; 0.535]	1.5%
Wang, B. 2017	121	465	0.260 [0.221; 0.303]	1.8%
Wang, H. 2020	55	226	0.243 [0.189; 0.305]	1.8%
Wu, Y. L. 2023	27	77	0.351 [0.245; 0.468]	1.6%
Yang. 2019	39	98	0.398 [0.300; 0.502]	1.6%
Yang, B. 2019	52	96	0.542 [0.437; 0.644]	1.6%
Yang, H. 2020	207	516	0.401 [0.359; 0.445]	1.8%
Yoshida. 2019	43	170	0.253 [0.190; 0.325]	1.7%
Yu, M. 2018	42	99	0.424 [0.325; 0.528]	1.6%
**Total (95% CI)**		**12800**	**0.294 [0.260; 0.327]**	**100.0%**

Heterogeneity: $Tau^2 = 0.016$; $Chi^2 = 1066.95$, df = 59 ($P < 0.01$); $I^2 = 94.47\%$

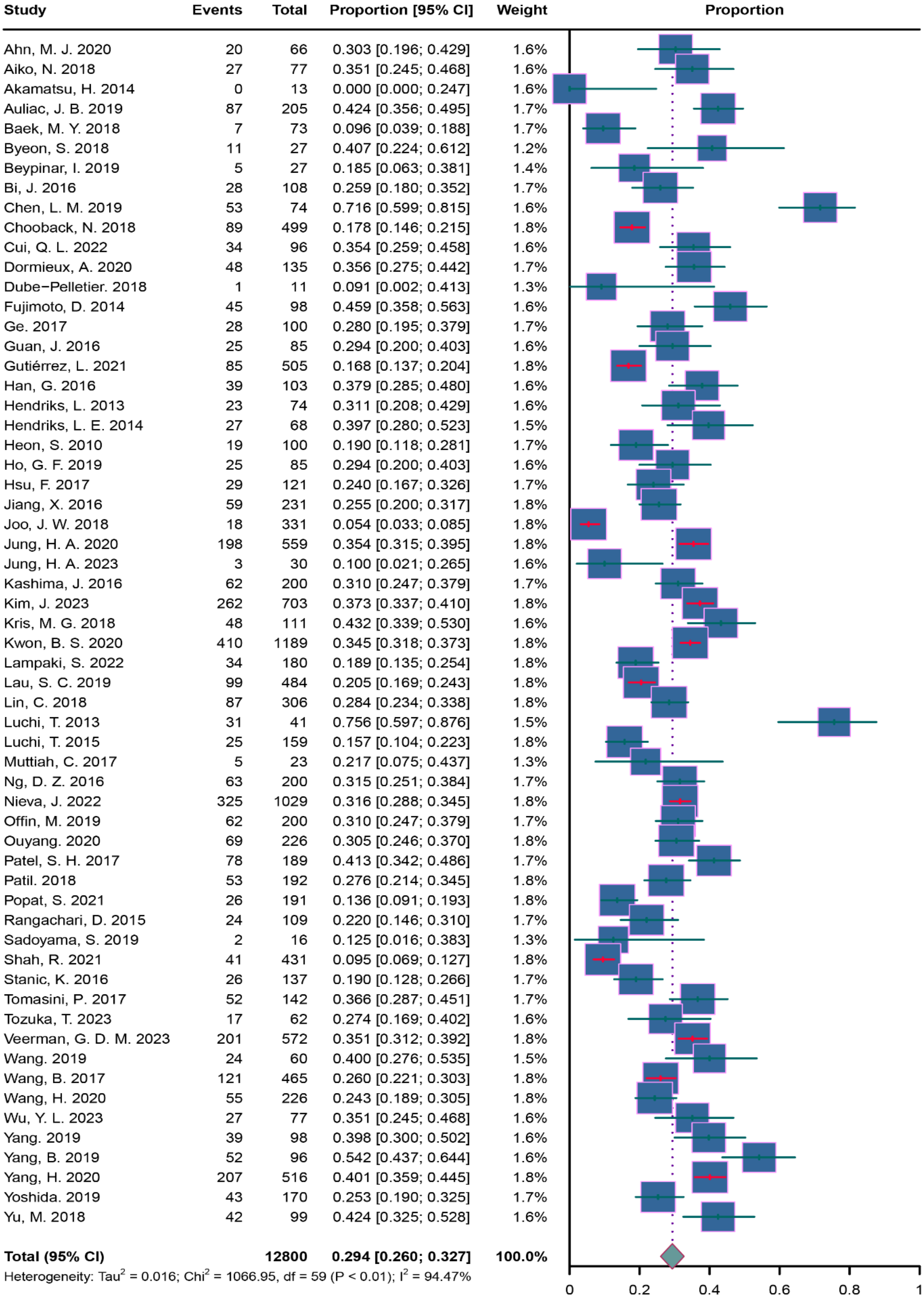

Supplementary Figure 2. Forest plot of brain metastases prevalence in ALK+ NSCLC (N=22).

Study	Events	Total	Proportion [95% CI]	Weight
Burudpakdee. 2018	49	181	0.271 [0.207; 0.342]	5.2%
Chu, L. 2019	27	40	0.675 [0.509; 0.814]	4.1%
Deng, H. 2019	12	47	0.255 [0.139; 0.403]	4.4%
Dormieux, A. 2020	34	81	0.420 [0.311; 0.535]	4.7%
Dube−Pelletier. 2021	2	3	0.667 [0.094; 0.992]	0.9%
Gadgeel. 2018	7	89	0.079 [0.032; 0.155]	5.3%
Gainor. 2017	77	196	0.393 [0.324; 0.465]	5.2%
Johung, K. L. 2016	27	90	0.300 [0.208; 0.406]	4.9%
Nakamura, T. 2023	12	42	0.286 [0.157; 0.446]	4.2%
Ni, J. 2019	35	93	0.376 [0.278; 0.483]	4.8%
Nishio, M. 2017	14	46	0.304 [0.177; 0.458]	4.3%
Ou, S. H. I. 2016	84	138	0.609 [0.522; 0.691]	5.0%
Rangachari, D. 2015	5	21	0.238 [0.082; 0.472]	3.5%
Patil MD. 2018	39	115	0.339 [0.253; 0.433]	5.0%
Shaw, A. T. 2020	38	149	0.255 [0.187; 0.333]	5.2%
Shi, Y. 2024	44	149	0.295 [0.223; 0.375]	5.1%
Solomon, B. J. 2022	78	296	0.264 [0.214; 0.318]	5.4%
Soria, J. C. 2017	121	376	0.322 [0.275; 0.372]	5.4%
Wang, H. 2020	4	22	0.182 [0.052; 0.403]	3.9%
Yang. 2019	3	16	0.188 [0.040; 0.456]	3.4%
Yang, H. 2020	24	76	0.316 [0.214; 0.433]	4.7%
Zhang, Y. 2020	21	112	0.188 [0.120; 0.272]	5.2%
**Total (95% CI)**		**2378**	**0.316 [0.259; 0.372]**	**100.0%**

Heterogeneity: $Tau^2 = 0.015$; $Chi^2 = 176.47$, df = 21 ($P < 0.01$); $I^2 = 88.10\%$

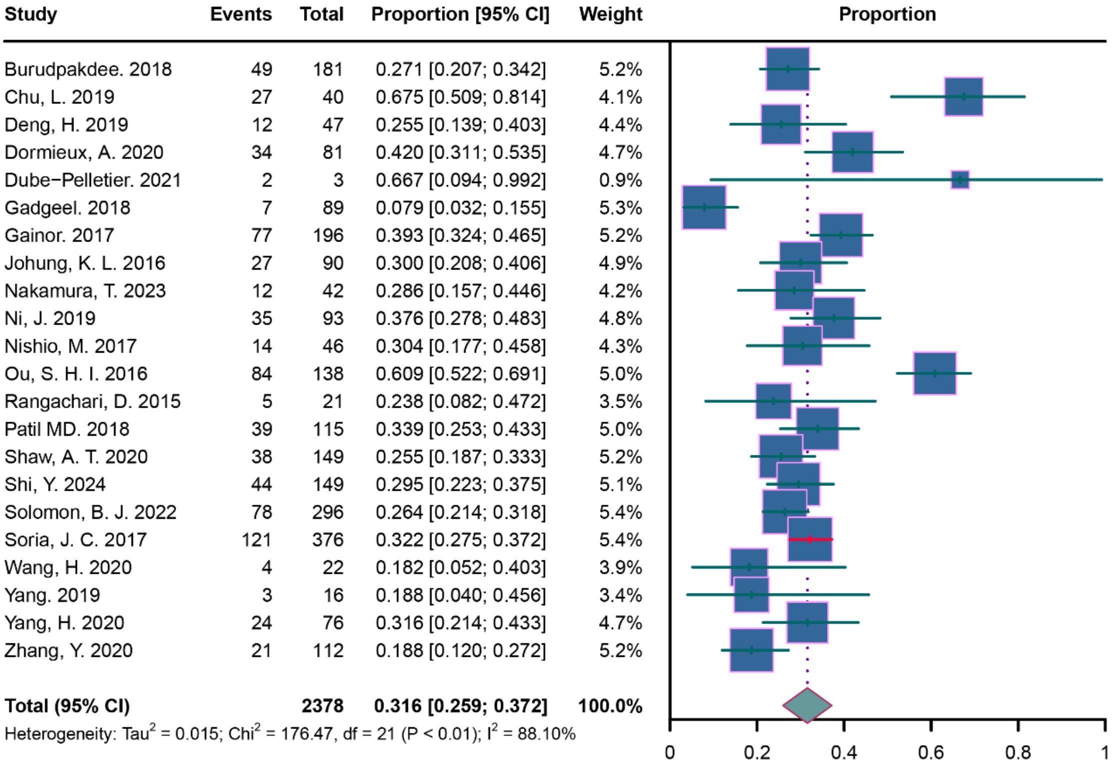

Supplementary Figure 3. Forest plot of brain metastases prevalence in KRAS+ NSCLC (N=11).

Study	Events	Total	Proportion [95% CI]	Weight
Calles. 2015	41	126	0.325 [0.245; 0.415]	9.6%
Dormieux, A. 2020	51	141	0.362 [0.283; 0.447]	9.7%
Hendriks, L. E. 2014	9	65	0.138 [0.065; 0.247]	9.5%
Kris, M. G. 2018	48	200	0.240 [0.183; 0.305]	10.5%
Noronha, V. 2024	17	133	0.128 [0.076; 0.197]	10.6%
Offin, M. 2019	48	200	0.240 [0.183; 0.305]	10.5%
Patil. 2018	29	102	0.284 [0.199; 0.382]	9.3%
Swart, E. M. 2023	30	153	0.196 [0.136; 0.268]	10.4%
Tomasini, P. 2016	18	47	0.383 [0.245; 0.536]	7.0%
Wang, H. 2020	6	55	0.109 [0.041; 0.222]	9.5%
Yang. 2019	6	13	0.462 [0.192; 0.749]	3.3%
**Total (95% CI)**		**1235**	**0.243 [0.185; 0.301]**	**100.0%**

Heterogeneity: $Tau^2 = 0.007$; $Chi^2 = 51.10$, df = 10 ($P < 0.01$); $I^2 = 80.43\%$

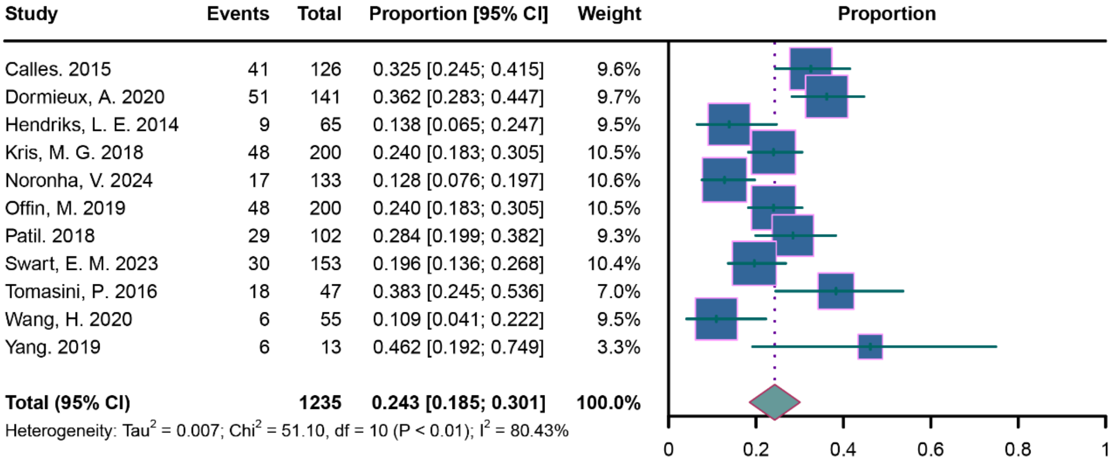

Supplementary Figure 4. Forest plot of brain metastases prevalence in ROS1+ NSCLC (N=6).

Study	Events	Total	Proportion [95% CI]	Weight
Ai, X. 2021	0	37	0.000 [0.000; 0.095]	21.8%
Gainor. 2017	10	39	0.256 [0.130; 0.421]	16.9%
Nakamura, T. 2023	3	26	0.115 [0.024; 0.302]	17.8%
Park, S. 2018	23	103	0.223 [0.147; 0.316]	20.1%
Patil. 2018	12	33	0.364 [0.204; 0.549]	15.3%
Wang, H. 2020	0	3	0.000 [0.000; 0.708]	8.1%
**Total (95% CI)**		**241**	**0.165 [0.049; 0.280]**	**100.0%**

Heterogeneity: $Tau^2 = 0.016$; $Chi^2 = 48.26$, df = 5 ($P < 0.01$); $I^2 = 89.64\%$

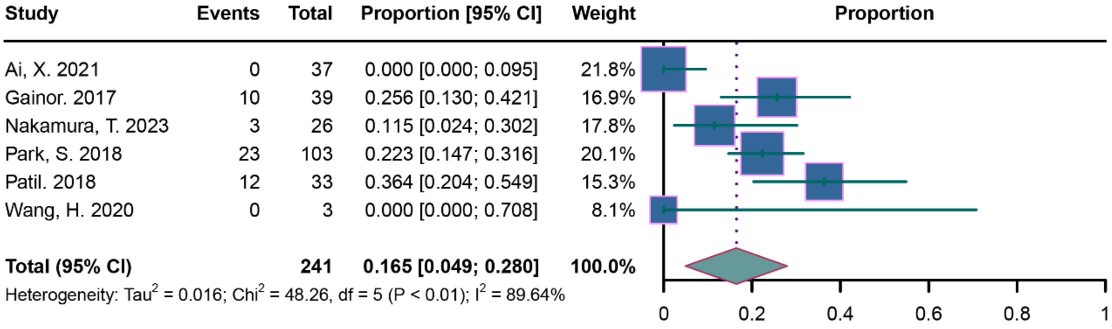

Supplementary Figure 5. Forest plot of brain metastases prevalence in RET+ NSCLC (N=4).

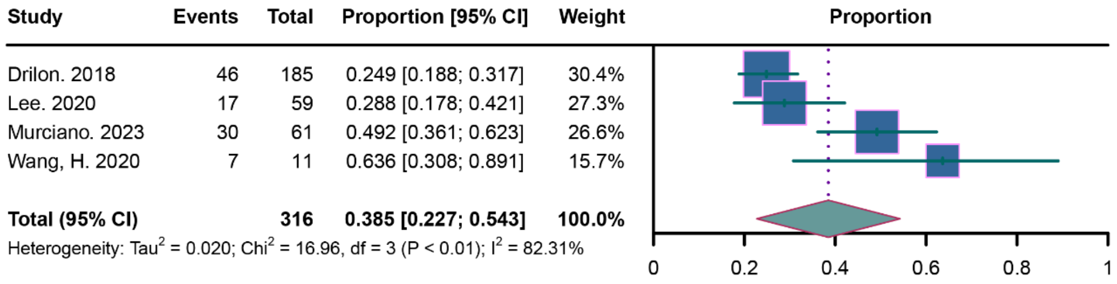

Supplementary Figure 6. Forest plot of brain metastases prevalence in HER2+ NSCLC (N=2).

Study	Events	Total	Proportion [95% CI]	Weight
Ahn, B. C. 2023	14	44	0.318 [0.186; 0.476]	39.2%
Offin. 2019	19	98	0.194 [0.121; 0.286]	60.8%
**Total (95% CI)**		**142**	**0.243 [0.124; 0.362]**	**100.0%**

Heterogeneity: $Tau^2 = 0.004$; $Chi^2 = 2.37$, df = 1 (P = 0.12); $I^2 = 57.77\%$

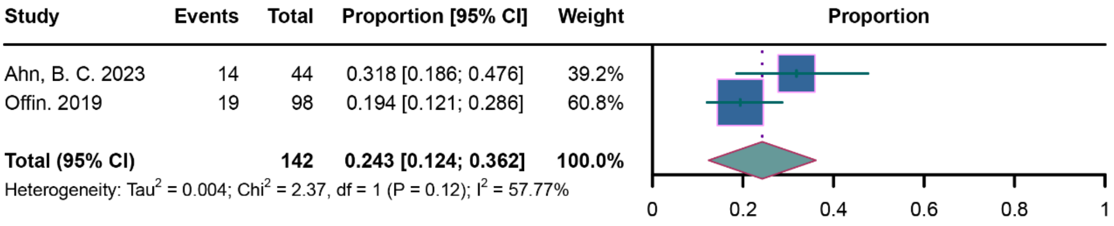

Supplementary Figure 7. Forest plot of brain metastases prevalence in MET+ NSCLC (N=2).

Study	Events	Total	Proportion [95% CI]	Weight
Offin, M. 2020	14	83	0.169 [0.095; 0.267]	94.2%
Wang, H. 2019	0	3	0.000 [0.000; 0.708]	5.8%
**Total (95% CI)**		**86**	**0.159 [0.081; 0.237]**	**100.0%**

Heterogeneity: $Tau^2 = 0$; $Chi^2 = 0.98$, df = 1 (P = 0.32); $I^2 = 0.00\%$

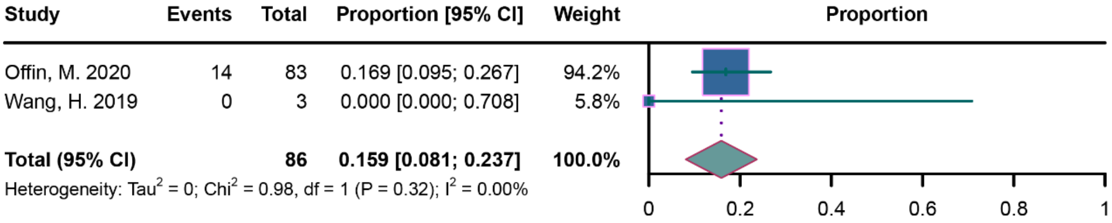

Supplementary Figure 8. Funnel plots for all forest plots generated.

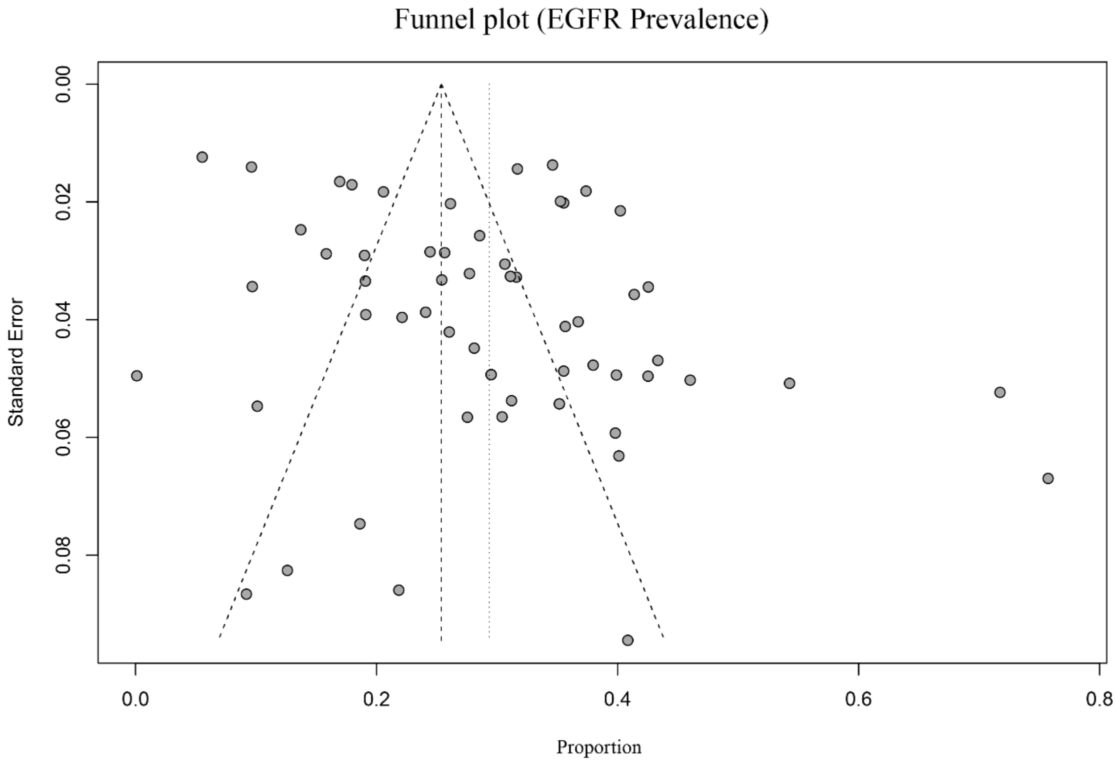

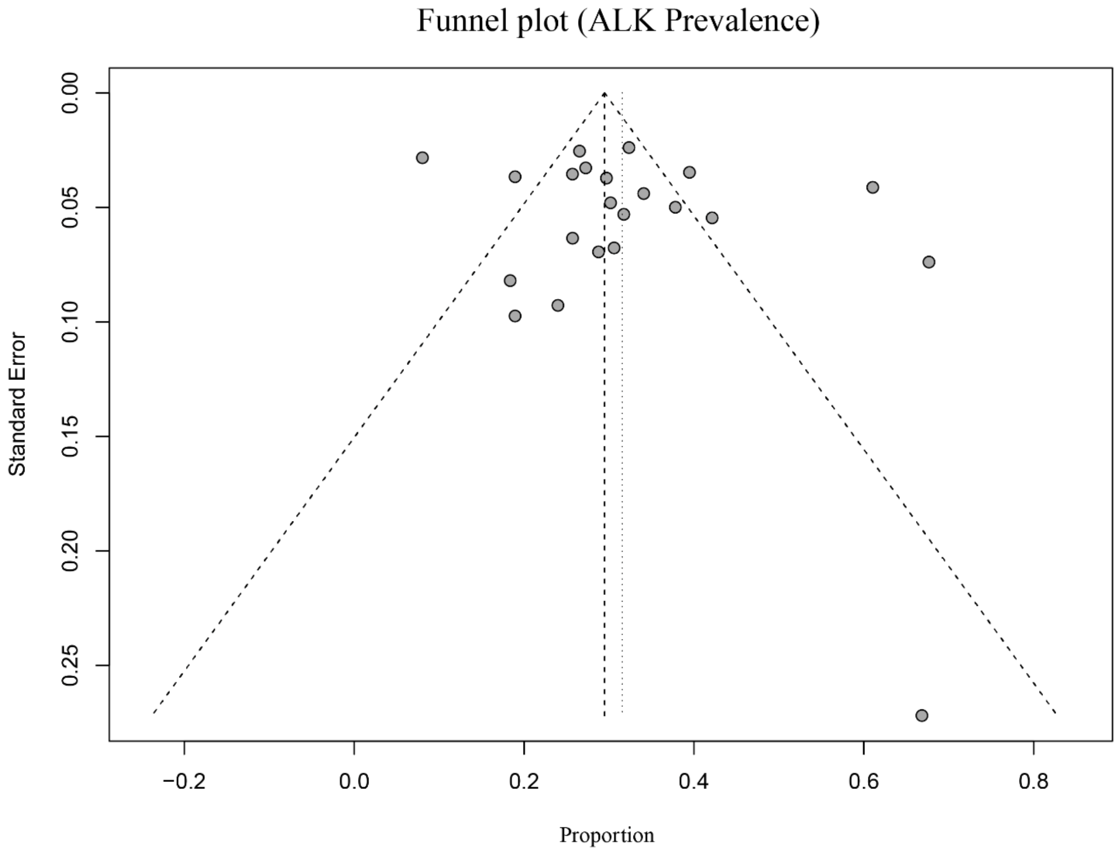

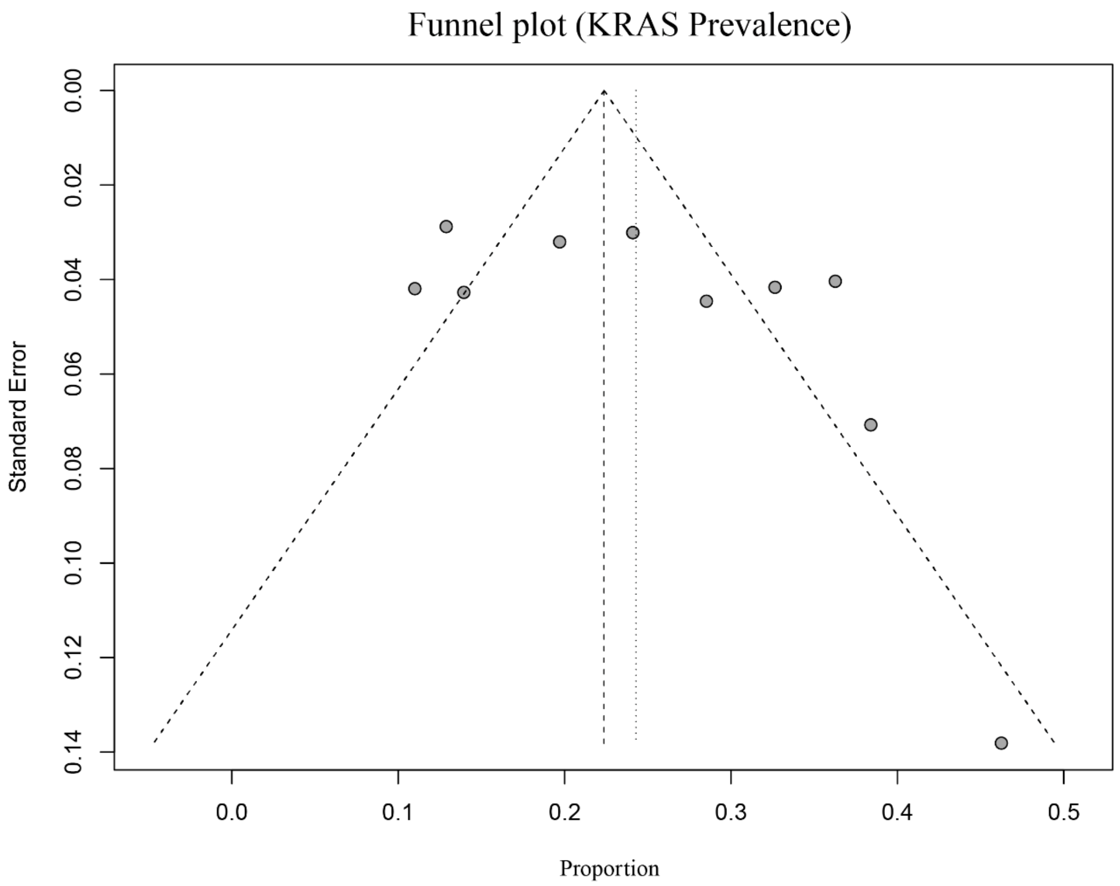

Funnel plot (KRAS Prevalence)
Standard Error
0.00
0.02
0.04
0.06
0.08
0.10
0.12
0.14
0.0
0.1
0.2
0.3
0.4
0.5
Proportion

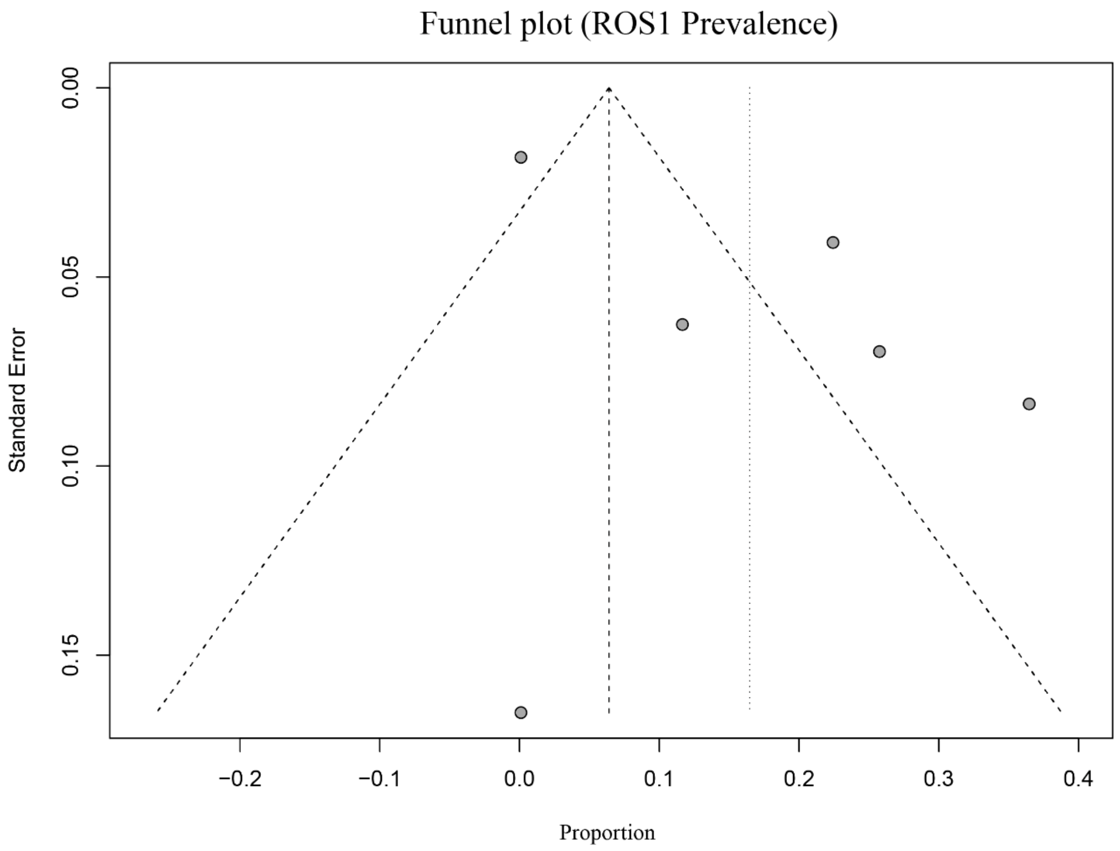

Funnel plot (ROS1 Prevalence)
Standard Error
0.00
0.05
0.10
0.15
−0.2
−0.1
0.0
0.1
0.2
0.3
0.4
Proportion

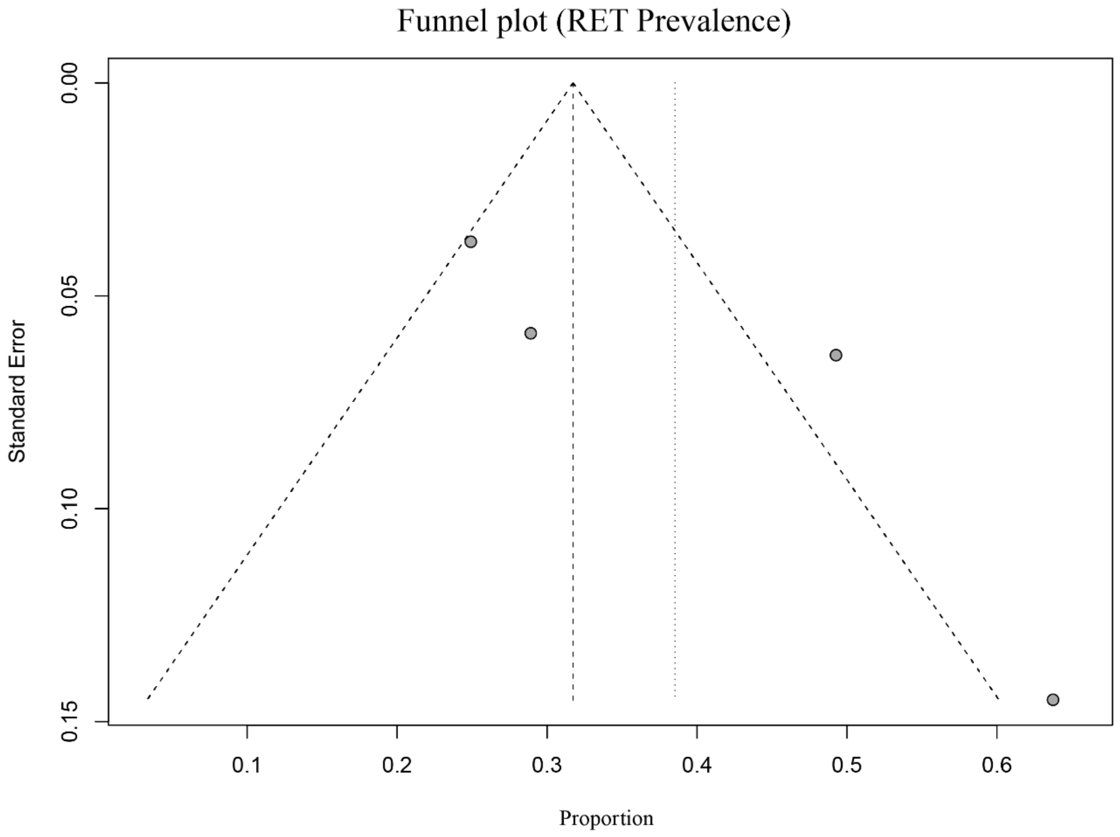
Funnel plot (RET Prevalence)
Standard Error
0.00
0.05
0.10
0.15
0.1
0.2
0.3
0.4
0.5
0.6
Proportion

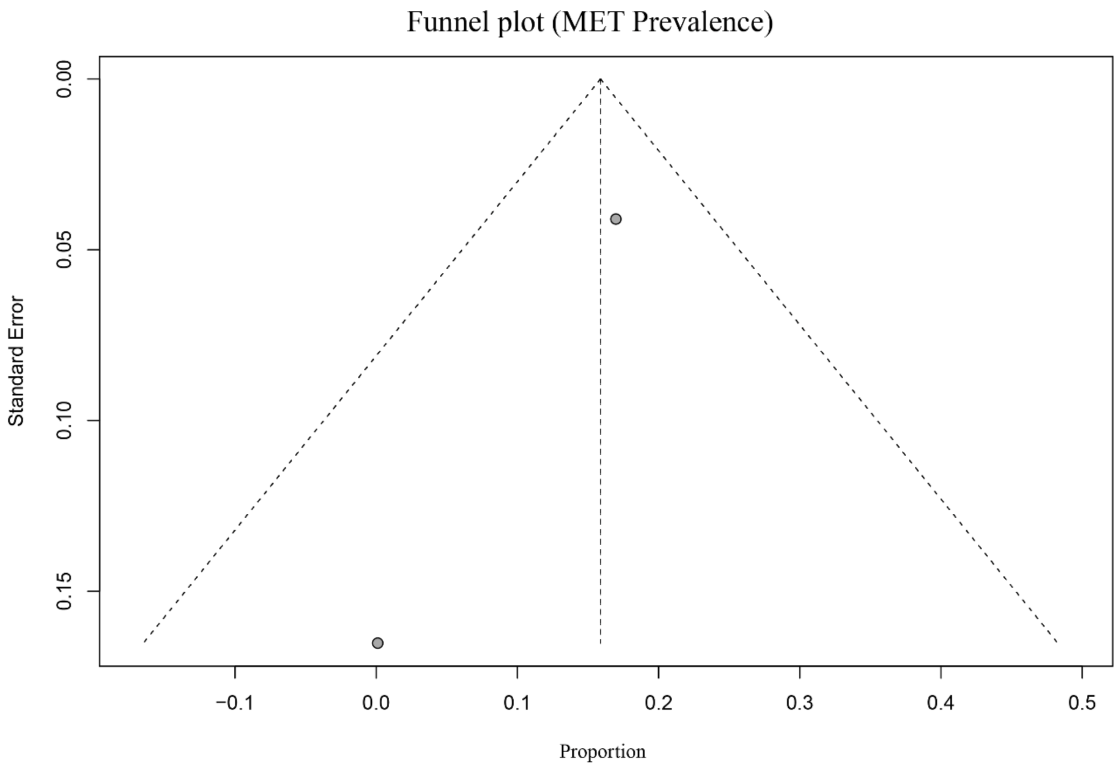
Funnel plot (MET Prevalence)
Standard Error
0.00
0.05
0.10
0.15
−0.1
0.0
0.1
0.2
0.3
0.4
0.5
Proportion

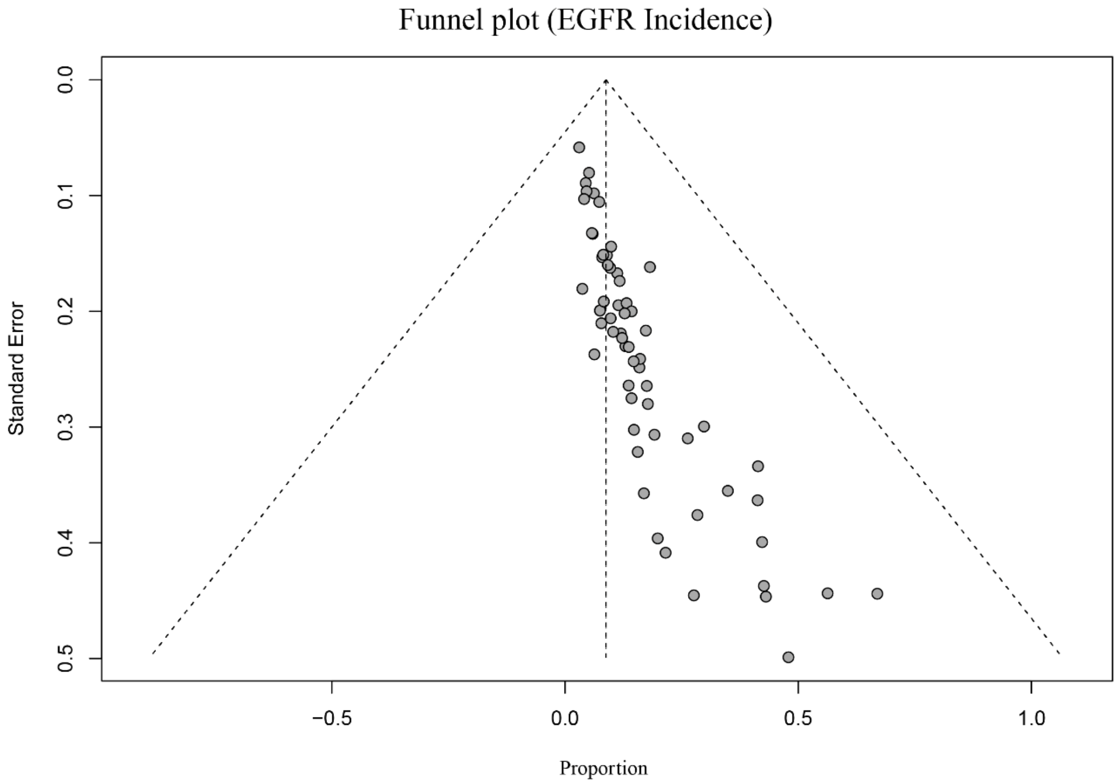
Funnel plot (EGFR Incidence)
Standard Error
0.0
0.1
0.2
0.3
0.4
0.5
−0.5
0.0
0.5
1.0
Proportion

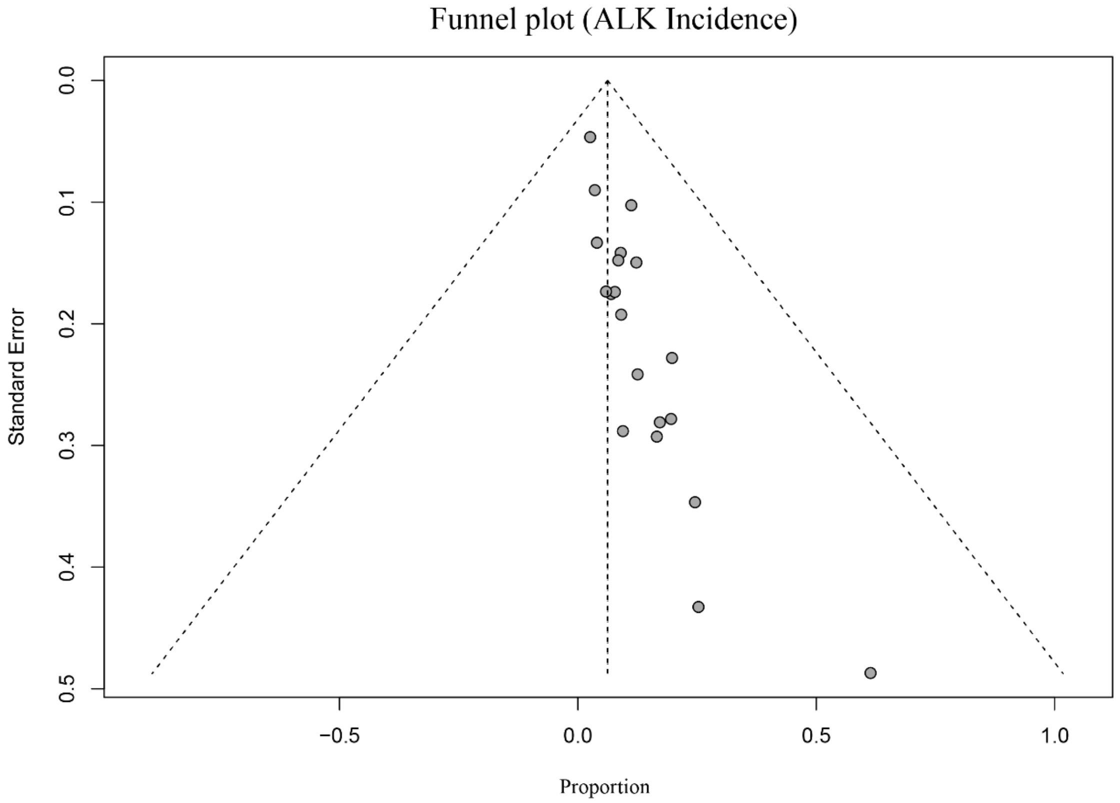
Funnel plot (ALK Incidence)
Standard Error
0.0
0.1
0.2
0.3
0.4
0.5
−0.5
0.0
0.5
1.0
Proportion

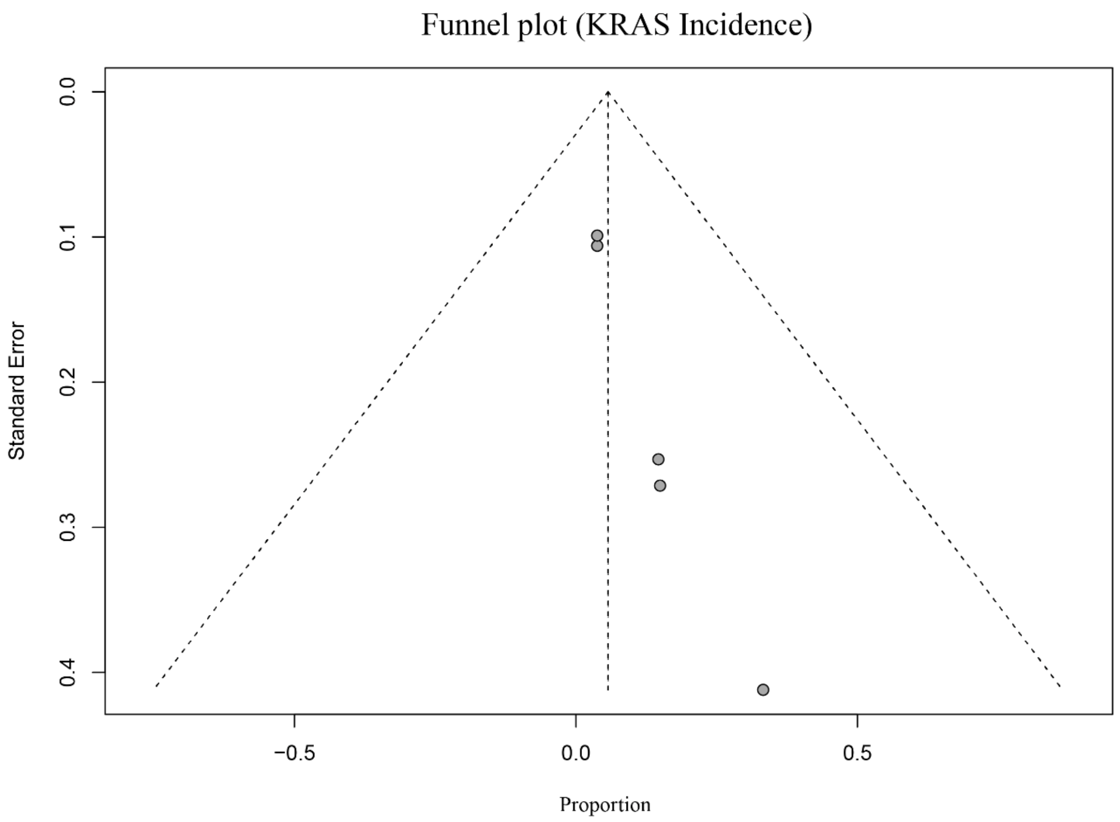

Funnel plot (KRAS Incidence)
Standard Error
0.0
0.1
0.2
0.3
0.4
−0.5
0.0
0.5
Proportion

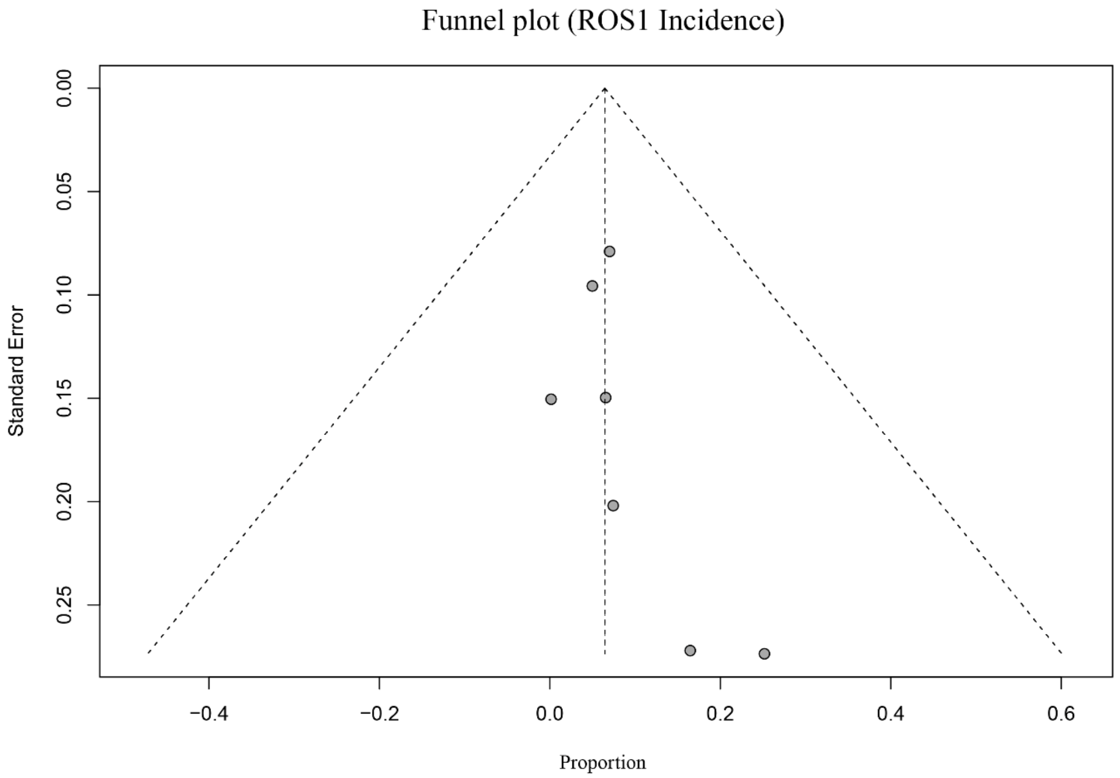

Funnel plot (ROS1 Incidence)
Standard Error
0.00
0.05
0.10
0.15
0.20
0.25
−0.4
−0.2
0.0
0.2
0.4
0.6
Proportion

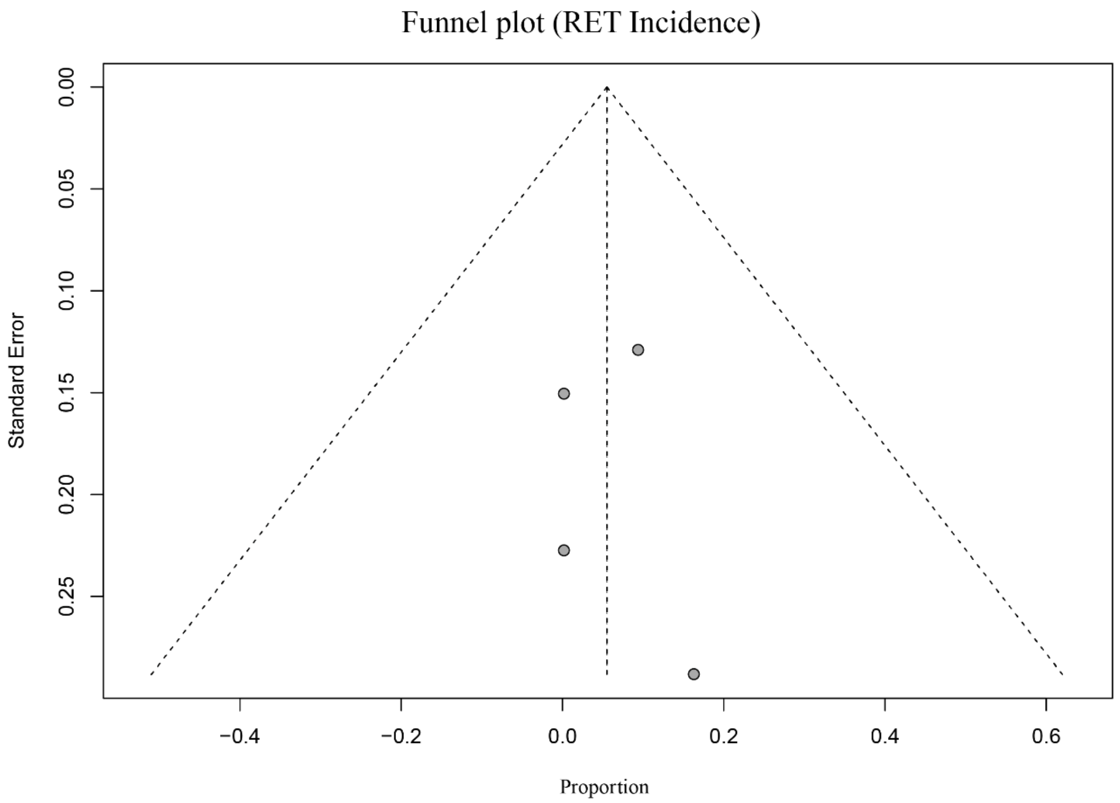
Funnel plot (RET Incidence)
Standard Error
0.00
0.05
0.10
0.15
0.20
0.25
−0.4
−0.2
0.0
0.2
0.4
0.6
Proportion

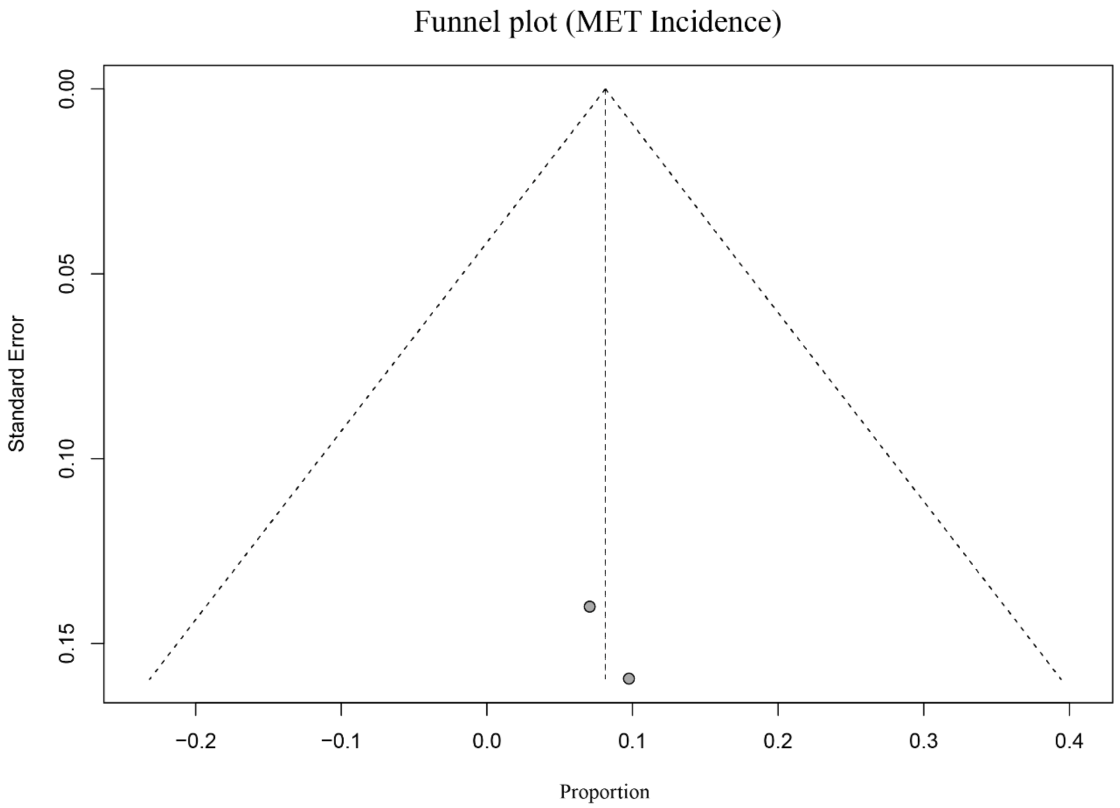
Funnel plot (MET Incidence)
Standard Error
0.00
0.05
0.10
0.15
−0.2
−0.1
0.0
0.1
0.2
0.3
0.4
Proportion

Supplementary Figure 9. Funnel plots for all forest plots generated.

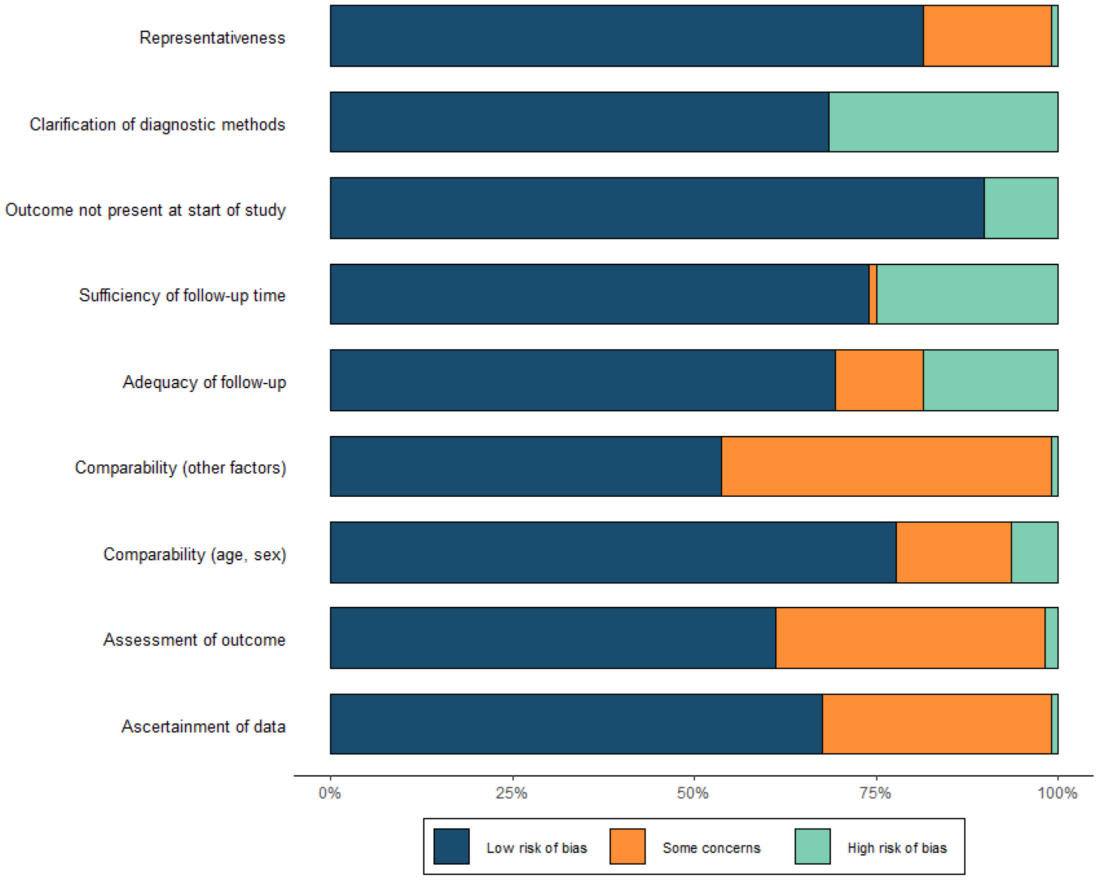

Supplement: Supplementary file 1 — Additional file 1 (PDF 2207 KB) [file 12672_2025_3621_MOESM1_ESM.pdf]
